# The DtxR protein acting as dual transcriptional regulator directs a global regulatory network involved in iron metabolism of *Corynebacterium glutamicum*

**DOI:** 10.1186/1471-2164-7-21

**Published:** 2006-02-09

**Authors:** Iris Brune, Hendrikje Werner, Andrea T Hüser, Jörn Kalinowski, Alfred Pühler, Andreas Tauch

**Affiliations:** 1 Institut für Genomforschung, Centrum für Biotechnologie, Universität Bielefeld, Universitätsstraße 25, D-33615 Bielefeld, Germany; 2 Lehrstuhl für Genetik, Fakultät für Biologie, Universität Bielefeld, Universitätsstraße 25, D-33615 Bielefeld, Germany

## Abstract

**Background:**

The knowledge about complete bacterial genome sequences opens the way to reconstruct the qualitative topology and global connectivity of transcriptional regulatory networks. Since iron is essential for a variety of cellular processes but also poses problems in biological systems due to its high toxicity, bacteria have evolved complex transcriptional regulatory networks to achieve an effective iron homeostasis. Here, we apply a combination of transcriptomics, bioinformatics, *in vitro *assays, and comparative genomics to decipher the regulatory network of the iron-dependent transcriptional regulator DtxR of *Corynebacterium glutamicum*.

**Results:**

A deletion of the *dtxR *gene of *C. glutamicum *ATCC 13032 led to the mutant strain *C. glutamicum *IB2103 that was able to grow in minimal medium only under low-iron conditions. By performing genome-wide DNA microarray hybridizations, differentially expressed genes involved in iron metabolism of *C. glutamicum *were detected in the *dtxR *mutant. Bioinformatics analysis of the genome sequence identified a common 19-bp motif within the upstream region of 31 genes, whose differential expression in *C. glutamicum *IB2103 was verified by real-time reverse transcription PCR. Binding of a His-tagged DtxR protein to oligonucleotides containing the 19-bp motifs was demonstrated *in vitro *by DNA band shift assays. At least 64 genes encoding a variety of physiological functions in iron transport and utilization, in central carbohydrate metabolism and in transcriptional regulation are controlled directly by the DtxR protein. A comparison with the bioinformatically predicted networks of *C. efficiens*, *C. diphtheriae *and *C. jeikeium *identified evolutionary conserved elements of the DtxR network.

**Conclusion:**

This work adds considerably to our currrent understanding of the transcriptional regulatory network of *C. glutamicum *genes that are controlled by DtxR. The DtxR protein has a major role in controlling the expression of genes involved in iron metabolism and exerts a dual regulatory function as repressor of genes participating in iron uptake and utilization and as activator of genes responsible for iron storage and DNA protection. The data suggest that the DtxR protein acts as global regulator by controlling the expression of other regulatory proteins that might take care of an iron-dependent regulation of a broader transcriptional network of *C. glutamicum *genes.

## Background

One of the major challenges in post-genomic research is to decipher and reconstruct the complete connectivity of transcriptional regulatory networks encoded by a bacterial genome sequence [1,2]. The key components in regulation of bacterial gene expression are DNA-binding transcription factors that are able to sense changing environmental conditions and to modulate the expression of relevant target genes. An important prerequisite for understanding the regulation of gene expression in a bacterial cell in its entirety is the identification of the repertoire of regulatory proteins encoded by a genome sequence along with the complete set of genes that are under transcriptional control by each of the identified regulators [3,4]. The transcriptional network of the Gram-negative bacterium *Escherichia coli *is currently the best-understood regulatory system of a single cell. It includes 314 DNA-binding transcription factors and is characterized by a hierarchical and modular architecture that is composed of eight modules with distinct physiological functions. Even for this well-studied model organism, only a small fraction of the transcriptional regulatory interactions are currently known [3,5].

The long-term objective of our post-genomic work is to characterize the complete repertoire of regulatory proteins of the Gram-positive bacterium *Corynebacterium glutamicum *along with the complete set of their target genes and corresponding DNA binding sites within the known genome sequence [6,7]. Since we also want to understand the evolution of the transcriptional regulatory network of *C. glutamicum*, we took advantage of the published genome sequences of *Corynebacterium efficiens *[8], *Corynebacterium diphtheriae *[9] and *Corynebacterium jeikeium *[10] to perform comparative genomic analyses. *C. glutamicum *and *C. efficiens *are widely used in biotechnological fermentation processes, whereas *C. diphtheriae *and *C. jeikeium *represent the most hazardous human pathogens among the corynebacteria. Therefore, the four species represent attractive targets to elucidate and compare not only the complete collection of DNA-binding transcriptional regulators but also the connectivity of regulatory interactions encoded by each genome sequence.

In a recent study, we have determined the complete sets of DNA-binding transcriptional regulators of the four corynebacteria and have performed a comparative content analysis of these genomes [7]. A collection of 127 DNA-binding transcriptional regulators was identified in the genome sequence of *C. glutamicum*, whereas 103 regulators were identified in *C. efficiens*, 63 in *C. diphtheriae *and 55 in *C. jeikeium*. The common set of transcriptional regulators encoded by the four corynebacterial genomes consists of only 28 proteins. Considering functional assignments deduced from computational predictions the common DNA-binding transcriptional regulators were grouped into five modules with distinct physiological functions [7]. The functional module "macroelement and metal homeostasis" includes, for instance, the transcriptional regulator McbR, directing the global regulation of almost all aspects of sulphur metabolism, a FurB homolog, most likely involved in regulation of zinc metabolism [7], and a transcriptional regulator that is homologous to the diphtheria toxin repressor DtxR of *C. diphtheriae *[11]. The *C. glutamicum *DtxR protein shares 71% identical amino acid residues with the orthologous counterpart of *C. diphtheriae*.

The diphtheria toxin repressor DtxR has been shown to be a global transcriptional regulator of iron metabolism in *C. diphtheriae *[12]. Iron is essential for a variety of cellular processes in virtually all organisms, since it plays important roles as enzyme cofactor or as integral part of cytochromes in oxidation-reduction and energy-generating systems [13]. On the other hand, iron poses problems in biological systems since the oxidized form is poorly soluble and the reduced form is highly toxic. Therefore, bacteria have evolved various mechanisms to counter these problems and to achieve an effective iron homeostasis. In addition, expression of the iron homeostatic machinery is subject to iron-dependent transcriptional regulation, for instance of genes encoding high-affinity iron uptake systems, intracellular iron storage proteins, redox-stress resistance systems, and iron-containing proteins to control overall iron consumption of the cell [13]. In *C. diphtheriae*, almost 20 binding sites of DtxR have been identified either by targeted genetic experiments or by a global repressor titration assay [14,15]. Major physiological functions that are controlled by DtxR in response to the iron level include the expression of the diphtheria toxin [12], the synthesis and export of siderophores [14], the siderophore-dependent uptake of iron [16], and the synthesis of specific systems for the utilization of iron from heme, hemin and hemoglobin [17,18].

In the present study, we have characterized the regulon of the transcriptional regulator DtxR of *C. glutamicum *by a genome-wide approach using DNA microarray technology. This global strategy along with bioinformatics predictions permitted the identification of DtxR binding sites in the *C. glutamicum *genome sequence along with a set of target genes that are under direct transcriptional control by DtxR. Binding of purified His-tagged DtxR protein to the upstream region of the respective genes was verified *in vitro *by DNA band shift assays. The resulting data were used for a comparative analysis of the deduced DtxR regulons in the four sequenced corynebacterial species.

## Results

### Phenotypic characterization of the dtxR mutant strain C. glutamicum IB2103

**Figure 1:**
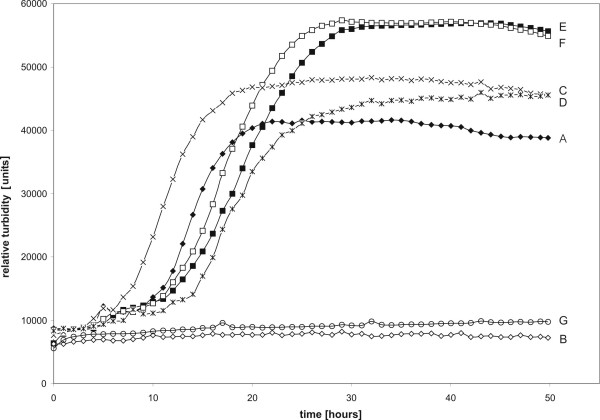
Growth curves of the wild-type strain *C. glutamicum *ATCC 13032 and the *dtxR *mutant *C. glutamicum *IB2103. The strains and plasmid-carrying derivatives were cultivated in CGXII medium and in low-iron CGXII medium, respectively. Growth was monitored with a nephelometer and is shown as relative turbidity. Values are means of measurements of six biological replicates. Abbreviations: A, *C. glutamicum *ATCC 13032 in CGXII medium; B, *C. glutamicum *IB2103 in CGXII medium; C, *C. glutamicum *ATCC 13032 in low-iron CGXII medium; D, *C. glutamicum *IB2103 in low-iron CGXII medium; E, *C. glutamicum *ATCC 13032 (pIB4000) in CGXII medium; F, *C. glutamicum *IB2103 (pIB4000) in CGXII medium; G, *C. glutamicum *ATCC 13032 (pEC-XK99E) in CGXII medium.

The *cg2103 *(*dtxR*) gene of *C. glutamicum *ATCC 13032 encodes a transcriptional regulator with significant amino acid sequence similarity to the diphtheria toxin repressor DtxR of *C. diphtheriae *[7,11]. To explore the regulatory network controlled by the DtxR protein of *C. glutamicum*, a defined deletion was established in the coding region of *dtxR *by applying a gene replacement strategy [19] that finally resulted in the mutant strain *C. glutamicum *IB2103. Subsequently, growth assays with *C. glutamicum *IB2103 were performed in liquid CGXII minimal medium and growth of the cultures was monitored by nephelometry (Figure 1). Growth assays with the wild-type strain *C. glutamicum *ATCC 13032 served as control. The growth assays clearly showed that *C. glutamicum *IB2103 containing the *dtxR *gene deletion was unable to grow in CGXII minimal medium. On the other hand, the *dtxR *mutant revealed the same growth characteristics as the wild-type strain when cultivated in low-iron CGXII medium (Figure 1). The growth deficiency of *C. glutamicum *IB2103 in CGXII minimal medium was complemented by transformation with plasmid pIB4000 carrying the cloned *dtxR *gene. Control assays with *C. glutamicum *IB2103 containing the empty cloning vector pEC-XK99E failed to complement the phenotype of the mutant (Figure 1). These data demonstrate that the defined deletion of *dtxR *in *C. glutamicum *IB2103 resulted in a conditionally lethal phenotype apparently depending on the amount of iron that is supplied with the synthetic culture medium. This distinct phenotype of *C. glutamicum *IB2103 was suited to perform comparative transcriptomic studies with the wild-type strain to identify genes that belong to the transcriptional regulatory network of DtxR.

### Genome-wide transcriptional profiling of the dtxR mutant C. glutamicum IB2103 in comparison to the wild-type strain ATCC 13032 by DNA microarray hybridization

To identify genes that are differentially expressed in the transcriptome of *C. glutamicum *IB2103, the global gene expression pattern of the *dtxR *mutant was compared with that of the wild-type strain by DNA microarray hybridization. Since *C. glutamicum *IB2103 was unable to grow in CGXII minimal medium (Figure 1), both strains were cultivated in shaking flasks to mid-exponential growth phase by using low-iron CGXII medium. The cultures revealed a very similar growth behavior that was characterized by almost identical doubling times of the *dtxR *mutant (4.8 ± 0.41 h) and the wild-type (4.4 ± 0.18 h), indicating that differences in growth of both strains were apparently minimized under the selected cultivation conditions. At the time when the *C. glutamicum *strains were grown to an optical density of four (OD_600 nm_), 10 mg/l FeSO_4 _was added to the growth medium, and the cultures were cultivated for further 15 min before harvesting cells and preparing total RNA. Addition of iron to the wild-type culture should result in a "switch-off" of expression of those genes that are part of the DtxR regulon, whereas the respective genes should be further on expressed in the *dtxR *mutant *C. glutamicum *IB2103. Therefore, genes revealing an enhanced expression in *C. glutamicum *IB2103 when compared to the wild-type are candidates that might belong to the transcriptional regulatory network of DtxR.

**Figure 2:**
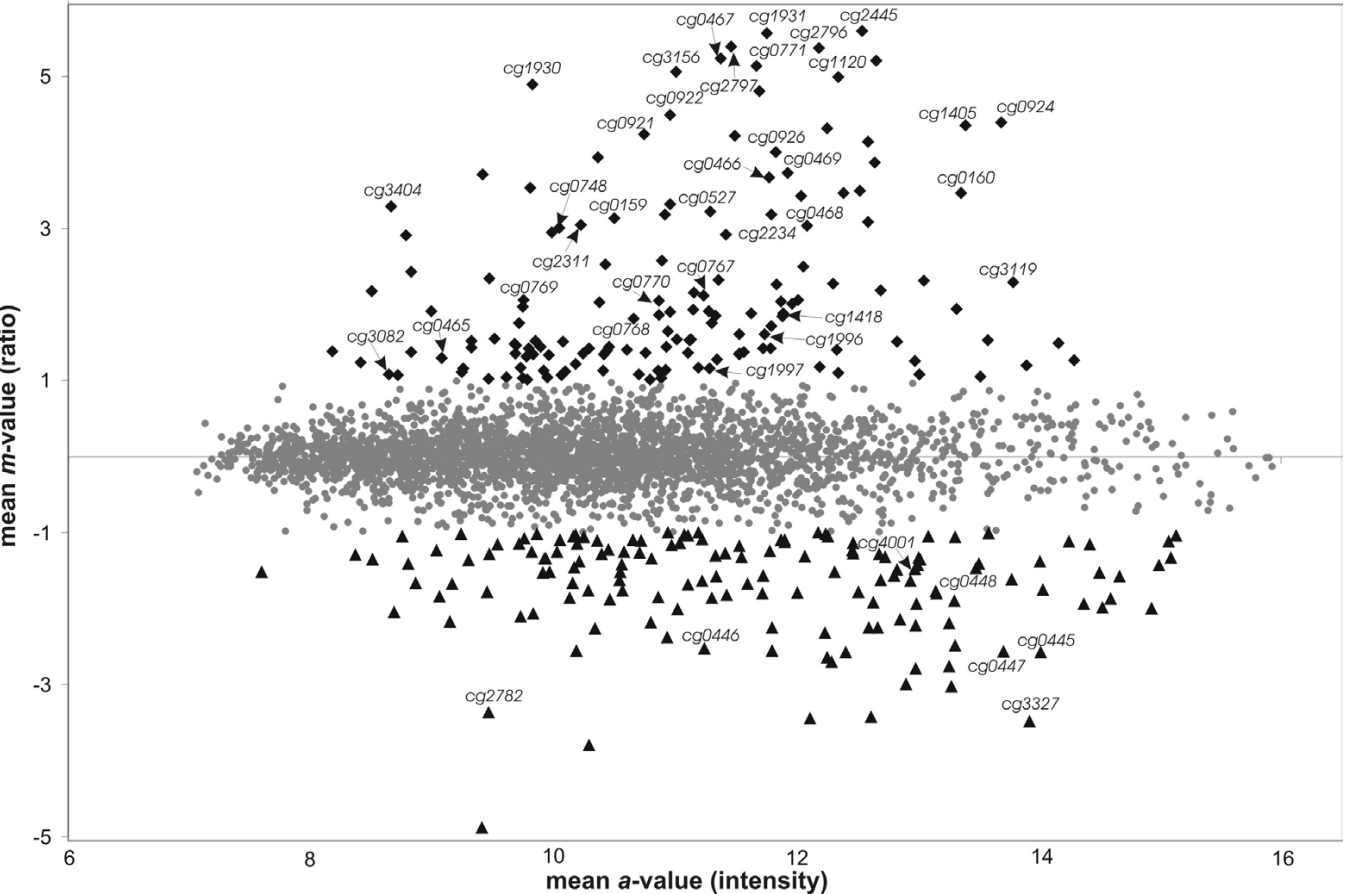
Ratio/intensity (*m*/*a*) plot deduced from DNA microarray hybridizations comparing the transcriptome of the *dtxR *mutant *C. glutamicum *IB2103 with that of the wild-type strain *C. glutamicum *ATCC 13032. The analyzed strains were cultivated in low-iron CGXII medium to mid-exponential growth phase. Then, 10 mg/l FeSO_4 _was added to the medium, and the cultures were incubated for further 15 min. Subsequently, total RNA was isolated from two biological replicates and used for DNA microarray hybridizations including label swapping. Genes showing enhanced expression in the *dtxR *mutant *C. glutamicum *IB2103 are marked by black diamonds; genes with decreased expression are indicated by black triangles; genes without differential expression pattern are shown by gray spots. Differentially expressed genes later on assigned to the DtxR regulon of *C. glutamicum *are named by their identifiers. Genes were regarded as being differentially expressed with *a*-values equal or greater than 7.0 and *m*-values equal or greater than 1 (up-regulation) or equal or smaller than -1 (down-regulation).

Cell samples for total RNA preparation were taken from two independently grown *C. glutamicum *cultures of each strain. The respective RNA preparations were used in two DNA microarray hybridization assays by applying label swapping. Labeling of probes and DNA microarray hybridization were carried out as described previously [20]. To minimize the number of false-positive signals, the data were stringently filtered to obtain genes with at least six statistically significant values out of the eight technical replicates present on the two microarrays along with an error probability of less than 5% for the Student's *t*-test. Normalization of the hybridization data by the LOWESS function and *t*-test statistics were accomplished by the EMMA software package. The resulting ratio/intensity (*m*/*a*) plot of the normalized data is presented in Figure 2.

By applying a ratio cut-off of ± 1, corresponding to relative expression changes equal or greater than twofold, a total of 257 genes revealed a differential expression in *C. glutamicum *IB2103 (see Additional file 1). This number includes 131 genes with significantly increased expression in *C. glutamicum *IB2103 when compared to the wild-type strain (*m*-value equal or greater than +1.0) and 126 genes with decreased expression in the *dtxR *mutant (*m*-value equal or smaller than -1.0). Several genes showing derepression of transcription in *C. glutamicum *IB2103 when compared to the wild-type strain encode iron-containing proteins and proteins that are apparently involved in iron transport and metabolism (Figure 2). Consequently, the upstream regions of the respective genes were ideally suited to search for the presence of a common DtxR binding motif.

### Computational identification of potential DtxR binding sites in the genome sequence of C. glutamicum ATCC 13032

To identify DtxR binding sites in the genome sequence of *C. glutamicum*, the upstream regions of 13 differentially expressed genes encoding proteins involved in iron metabolism were selected (Table 1) and aligned by using the CLUSTAL X program [21]. The alignment identified a common 19-bp DNA motif with the palindromic consensus sequence 5'-TTAGGTTAG(G/C)CTAACCTAA-3' that is identical to DtxR binding sites identified earlier in *C. diphtheriae *[14]. A CLUSTAL X alignment of the 19-bp motifs was used to create a Hidden Markov model (HMM), and the *C. glutamicum *genome sequence was searched for the presence of additional motifs with this HMM profile. The results generated by HMM searches were then compared with the genomic positions of genes that showed differential expression in the *dtxR *mutant *C. glutamicum *IB2103 during DNA microarray hybridization. This bioinformatics approach identified 24 motifs in front of genes that were identified as differentially expressed by DNA microarray hybridization, including the 13 motifs initially used as seed information to create the HMM profile (Table 1). It is noteworthy that three 19-bp motifs were predicted in front of genes (*cg2782*/*ftn*, *cg3327*/*dps*, *cg0445*/*sdhCD*) that showed a decreased expression in the *dtxR *mutant *C. glutamicum *IB2103 (Table 1). The bioinformatics search identified additional 16 motifs upstream of genes that were not detected during the DNA microarray hybridization experiment. These motifs either represent false-positive predictions or might be related to genes that were not detected with significant values during DNA microarray hybridization (see below). Accordingly, a total number of 40 potential DtxR binding sites was identified in the *C. glutamicum *genome sequence by bioinformatics analysis and further on investigated experimentally. Especially genes that showed an enhanced or decreased expression in the *dtxR *mutant *C. glutamicum *IB2103 and that are moreover characterized by the presence of a predicted binding site within the corresponding upstream region can be considered as candidates for direct transcriptional regulation by DtxR.

**Table 1:** Identification of DtxR binding sites in the genome sequence of *C. glutamicum*

CDS^1^	Gene	Predicted DtxR binding motif^2^	Distance to translational start codon^3^	Differential gene expression in *C. glutamicum *IB2103 measured by	
				
				DNA microarray (ratio)^4^	Real-time RT-PCR (relative expression)^5^
cg0466*	-	TTAAGTTAGCATAGCCTTA	141	+3.2	+1160
cg0748*	-	ATAGGATAGGTTAACCTGA	25	+3.0	+29
cg0771*	-	GTCGGGCAGCCTAACCTAA	40	+5.3	+239
cg0922*	-	TAAGGTTTGCCTAATCTTT	30	+4.4	+176
cg0924*	-	TTAGGTAACCTAACCTCAC	63	+4.4	+2000
cg0926*	-	TTAGGTTAGGCTCTAATAT	173	+4.0	+185
cg1405*	-	TTTTGTTAGGCTTGCCTAG	33	+4.3	+198
cg1418*	-	TTAGGTAAGGTTTGCATAC	30	+1.8	+119
cg2234*	-	TTAGGCAAGGCTACCTTTT	4	+3.2	+1980
cg2445*	*hmuO*	GTAGGTGTGGGTAACCTAA	120	+5.6	+203
cg2782*	*ftn*	TTATGCTGCGCTAACCTAT	37	-3.3	+0.02
cg3327*	*dps*	TCAGGATAGGACAACCTAA	61	-3.4	+0.01
cg3404*	-	TTAGGCTATCCTAACGCAA	overlap	+3.2	+364
*cg0160*	-	AATGGTTAGGCTAACCTTA	overlap	+3.4	+1870
*cg0445*	*sdhCD*	TAAAGTAAGGCTATCCTAA	111	-2.5	+0.12
*cg0527*	-	TTAGGCTTGCCATACCTAT	11	+3.2	+63
*cg1120*	*ripA*	TGAGGTTAGCGTAACCTAC	32	+4.9	+359
*cg1930*	-	TTAGGTAAAGCTTGCCTAT	90	+4.8	+1210
*cg1996*	*cglIM*	TTAGGATTCTCTCAACTAA	199	+1.7	+3.8
*cg2311*	-	TCAAGTAAGGTTTACCTTA	overlap	+3.0	+37
*cg2796*	-	GAAGGCAAGCCAAACTTAA	18	+5.3	+1610
*cg3082*	-	TTCTGTGAGGTTAACTTTT	254	+1.1	+19
*cg3119*	*fpr2*	TTAGGTTAGGTTCACCGTG	204	+2.2	+18
*cg3156*	-	TAAGGCAAGCCTAAATTAG	97	+5.0	+304
*cg0405*	-	TAAGGATAACCTTGCCTTA	28	n.d.	+1010
*cg0470*	-	TTAGGTTAAGCTAATCTAG	32	n.d.	+978
*cg0591*	-	TTAGTAAAGGCTCACCTAA	91	n.d.	+310
*cg0955*	-	TTACGTGAGCGTAGCCGAA	200	n.d.	+2.8
*cg1642*	-	TTAGGTTAGGCAAGCCATA	39	n.d.	+4.4
*cg3247*	*cgtR11*	ATCAGTAAGGCTAGACTAA	87	n.d.	+5.5
*cg3394*	-	TTAAGGTAAGTTCAGCTAA	37	n.d.	+4.6
*cg1070*	-	ATAGGTTATCCAAGCCTAA	18	n.d.	n.d.
*cg1088*	-	TTAAGTCAGTGTTACCTAA	82	n.d.	n.d.
*cg1966*	-	TAACTTTGACATAACCTAA	67	n.d.	n.d.
*cg2224*	*xerC*	GTGTGTGAGGCAAGCCTAA	93	n.d.	n.d.
*cg2399*	*glk*	AAAGATTAAATTCACCTAA	29	n.d.	n.d.
*cg2598*	-	TTAGGTCAAGCTTGCATTT	20	n.d.	n.d.
*cg2651*	-	TTAGGTTGTAAAAACCTTA	46	n.d.	n.d.
*cg2842*	*phoU*	TTAGGTGATTCAATCTTAA	50	n.d.	n.d.
*cg3170*	-	TTAACTTTGCCCTACCTAA	190	n.d.	n.d.

^1 ^Asterisks denote genes whose upstream regions were used for initial HMM processing.

^2 ^Bases belonging to predicted translational start codons are underlined.

^3 ^The distance from the last nucleotide of the DtxR binding site to the translational start codon is indicated.

^4 ^n.d., not detected as differentially expressed by using a significance cut-off of ± 1 (ratio).

^5 ^n.d.; not detected as differentially expressed by using a significance cut-off of +2 (up-regulation) or +0.5 (down-regulation).

### Verification of differential expression of genes with predicted DtxR binding sites by real-time RT-PCR experiments

Differential expression of the 40 genes identified by bioinformatics screening of the *C. glutamicum *genome sequence for the presence of DtxR binding sites was examined further by real-time RT-PCR assays using the same experimental setup as applied for DNA microarray hybridizations. The resulting data are summarized in Table 1. The real-time RT-PCR assays demonstrated that the expression of 31 genes was enhanced up to 2000-fold in the *dtxR *mutant *C. glutamicum *IB2103 when compared with the wild-type strain. Accordingly, the results can be divided into three categories: (i) Differential expression in *C. glutamicum *IB2103 of the 24 genes initially identified by the DNA microarray experiment was confirmed by the RT-PCR approach. (ii) Among the genes additionally predicted by HMM searches seven showed indeed a differential expression in the *dtxR *mutant strain *C. glutamicum *IB2103. Expression of four genes was enhanced only up to 5-fold, and a further examination of the DNA microarray data indicated that at least two of the respective ratios were close to the experimental cut-off (data not shown). (iii) In the case of nine potential motifs, the prediction by the HMM profile was most likely false-positive, since the corresponding genes showed no significant changes of the expression level in *C. glutamicum *IB2103, neither by DNA microarray hybridization nor by real-time RT-PCR measurements. Consequently, the combination of DNA microarray hybridization, bioinformatics screening approaches and real-time RT-PCR assays led to a set of genes that are differentially expressed in the *dtxR *mutant *C. glutamicum *IB2103 and that are characterized by a putative DtxR binding site within the upstream region.

### Binding of purified DtxR protein to the identified sequence motifs as analyzed by DNA band shift assays

To demonstrate binding of the DtxR regulator to the predicted 19-bp motifs by DNA band shift assays, a modified DtxR protein was constructed and subsequently purified. For this purpose, the coding region of the *dtxR *gene was fused with a 3' extension encoding six histidine residues and amplified by PCR. The resulting DNA fragment was cloned in *E. coli *into the expression vector pEC-XK99E [22] resulting in plasmid pIB4001 that is suitable for heterologous overexpression of the modified DtxR protein upon IPTG induction. The His-tagged DtxR protein was purified to homogeneity from an induced *E. coli *culture by means of Ni-TED columns and positively identified by MALDI-TOF mass spectrometry and peptide mass fingerprinting (data not shown). Moreover, plasmid pIB4001 was transferred into the *dtxR *mutant *C. glutamicum *IB2103 by electroporation to prove that the His-tagged DtxR protein is functional *in vivo*. This control experiment demonstrated that the growth deficiency of the *dtxR *mutant *C. glutamicum *IB2103 in CGXII minimal medium can be complemented by plasmid pIB4001 but not by the empty cloning vector pEC-XK99E (data not shown).

**Figure 3:**
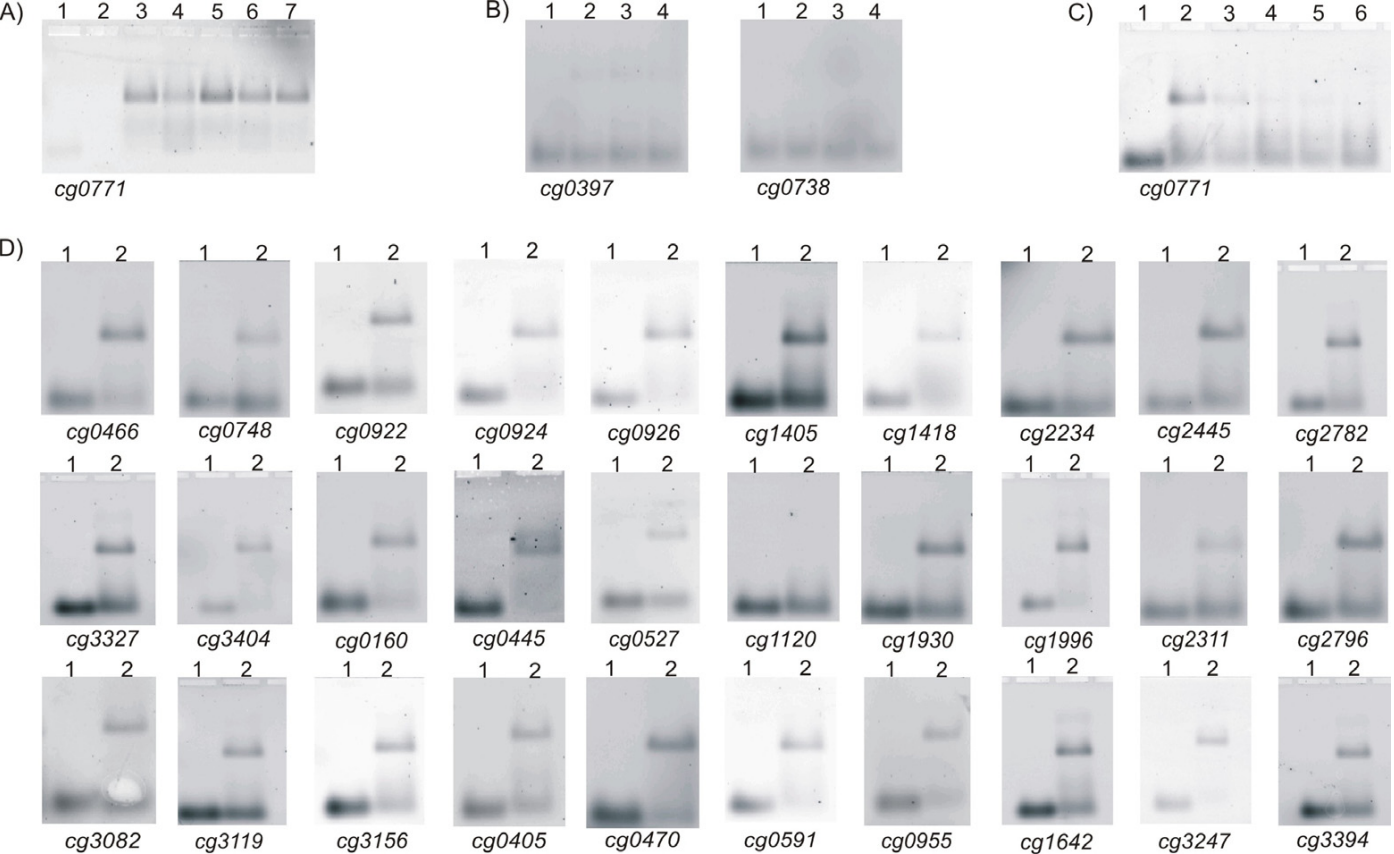
Agarose gels of DNA band shift assays performed with purified His-tagged DtxR protein. (A) DNA band shift assays with a Cy3-labeled double-stranded 40 mer covering the predicted DtxR binding sites in front of the *cg0771 *gene. Band shifts were performed with 0.05 pmol of the labeled 40 mer DNA fragment and different amounts of the His-tagged DtxR protein. Assays were separated in a 2% agarose gel and visualized by fluorescence imaging. Lane 1: control assay without DtxR protein; lane 2: control assay without 40 mer; lane 3: band shift assay with 42 pmol DtxR; lane 4: assay with 84 pmol DtxR; lane 5: assay with 126 pmol DtxR; lane 6: assay with 168 pmol DtxR; lane 7: assay with 210 pmol DtxR. (B) Control experiments with Cy3-labeled 40 mers deduced from internal gene regions of *cg0397 *and *cg0738 *(*dnaE2*). Lanes 1: control assay without DtxR protein; lanes 2: control assay containing 42 pmol DtxR protein; lanes 3: assays with 84 pmol DtxR; lanes 4: assays with 126 pmol DtxR. (C) DNA displacement experiments with a Cy3-labeled double-stranded 40 mer covering the predicted DtxR binding sites in front of the *cg0771 *gene. During displacement studies, 42 pmol of purified His-tagged DtxR protein and 0.05 pmol of a Cy3-labeled 40 mer along with increasing concentrations of the same non-labeled 40 mer fragment were added to assay. Lane 1: control assay without purified DtxR protein; lane 2: control assay without non-labeled 40 mer; lane 3: assay with 0.015 pmol non-labeled 40 mer; lane 4: assay with 0.3 pmol non-labeled 40 mer; lane 5: assay with 0.45 pmol non-labeled 40 mer; lane 6: assay with 1 pmol non-labeled 40 mer. (D) Verification of the predicted DtxR binding sites by DNA band shift assays using 0.05 pmol of Cy3-labeled 40 mers and 42 pmol of purified His-tagged DtxR protein. Gene identifiers are shown below the agarose gels. Lanes 1: control assay without DtxR protein; lanes 2: DNA band shift assay containing DtxR protein.

Subsequently, DNA band shift assays were carried out with the purified His-tagged DtxR protein and distinct DNA fragments containing each of the 31 predicted 19-bp motifs. For this purpose, the motifs were extended on both sides with the respective gene-specific sequences to a total length of 40 nucleotides. These 40 mer oligonucoletides were labeled with Cy3 and annealed to produce double-stranded DNA fragments that were applied in band shift assays. First of all, 0.05 pmol of the labeled 40 mer DNA fragment representing the predicted DtxR binding site upstream of *cg0771 *was incubated with different amounts of His-tagged DtxR protein (42 pmol, 84 pmol, 126 pmol, 168 pmol, and 210 pmol). The assays were then separated by agarose gel electrophoresis and visualized by fluorescence imaging (Figure 3A). Apparently, the presence of purified DtxR protein caused a different electrophoretic mobility of the labeled DNA fragment even at the low protein concentration of 42 pmol. Furthermore, DNA band shift assays were performed with labeled 40 mer control fragments located within the coding region of *cg0397 *and *cg0738 *(*dnaE2*), respectively. Incubation of purified DtxR protein with these DNA fragments delivered no band shift (Figure 3B). Binding of the DtxR protein to the upstream region of *cg0771 *was also analyzed by DNA displacement experiments, in which increasing concentrations of the same non-labeled 40 mer fragment (0.015 to 1 pmol) were added to the band shift assay. Increasing concentrations of this sequence-specific competitor prevented the DNA band shift caused by the purified DtxR protein (Figure 3C). These data suggested a specific binding of the DtxR regulator to the upstream region of *cg0771 *containing the common 19-bp motif.

Subsequent DNA band shift assays were performed with 42 pmol of purified DtxR protein (Figure 3D). The presence of purified DtxR protein caused a band shift of almost all tested 40 mer DNA fragments, with exception of a 40 mer region located in front of the *cg1120 *gene that encodes the ArsR-type regulatory protein RipA [23]. The reason for this observation is currently unknown since binding of the DtxR protein to the upstream region of *cg1120 *was described earlier [23]. Nevertheless, the DNA band shift assays confirmed *in vitro *the binding of the DtxR regulator to specific DNA fragments containing the common 19-bp motif that was identified in the upstream region of differentially expressed genes.

### Localization of DtxR binding sites in mapped promoter regions of differentially expressed genes

**Figure 4:**
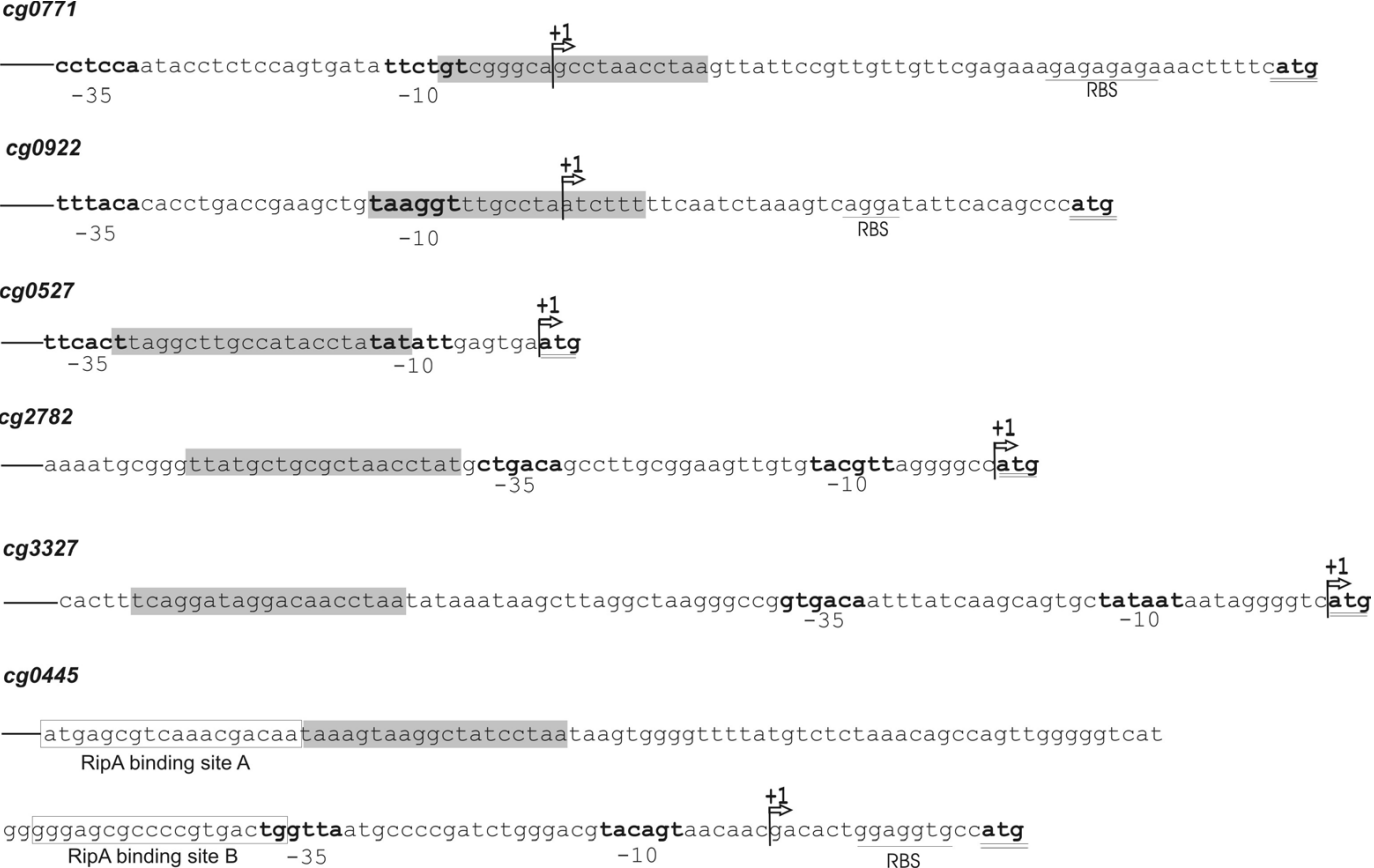
Detailed genetic maps of upstream regions of selected genes belonging to the DtxR regulon of *C. glutamicum*. The nucleotide sequences shown indicate the DtxR binding sites (gray boxes) and the identified transcriptional start sites that were mapped with the RACE method (+1). Bold-faced nucleotides display deduced -35 and -10 hexamer sequences of corynebacterial promoter regions. Potential ribosome-binding sites (RBS) in front of the coding regions are underlined; the ATG start codon is double underlined. The *cg0527*, *cg2782 *and *cg3327 *genes are expressed by leaderless transcripts. Previously identified RipA binding sites in front of the *cg0445 *genes are marked [23].

To determine the position of verified DtxR binding sites in relation to the promoter region of the respective genes, transcriptional start sites were identified by using the 5' RACE method. Transcriptional starts were determined for the genes *cg0771*, *cg0922 *and *cg0527 *that showed an enhanced expression in the *dtxR *mutant *C. glutamicum *IB2103 and for the *cg2782*, *cg3327 *and *cg0445 *genes that revealed a decreased expression. The detected transcriptional start sites were identified in variable distances to the annotated translational starts of the corresponding proteins (Figure 4). The transcriptional start sites of *cg0527*, *cg2782 *and *cg3327 *were identical to the adenine residue of the annotated translational start codons. These results provided evidence for the presence of leaderless transcripts that are known to occur in *C. glutamicum *[24]. Moreover, the determination of transcriptional start points provided the basis for the identification of potential -35 and -10 promoter regions, according to the known features of corynebacterial promoter sequences [24]. At least three nuclotides of the potential -35 and -10 hexamers were identical to the consensus sequences TTGCCA and TA(C/T)AAT, respectively. In the case of *cg0771*, *cg0922 *and *cg0527 *the verified DtxR binding sites were located within the deduced promoter region (Figure 4), which is consistent with the physiological role of the DtxR protein as transcriptional repressor of gene expression. On the other hand, the DtxR binding sites of *cg2782*, *cg3327 *and *cg0445 *were located upstream of the deduced -35 promoter regions (Figure 4). Since expression of these genes decreased in the *dtxR *mutant *C. glutamicum *IB2103, as measured by DNA microarray hybridization and real-time RT-PCR, the location of the DtxR binding sites would be more consistent with an activating function of the DtxR protein [25].

### Bioinformatics characterization and functional dissection of the DtxR regulon of C. glutamicum

**Figure 5:**
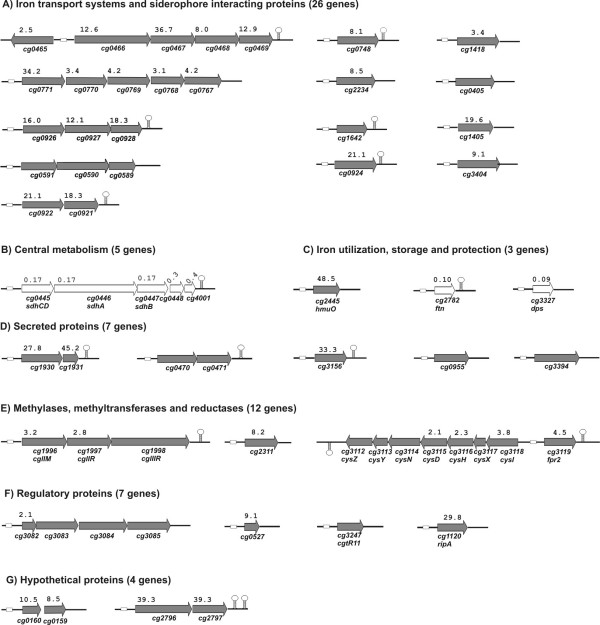
Functional dissection of genes belonging to the DtxR regulon of *C. glutamicum*. Genes and gene clusters showing differential expression in the *dtxR *mutant *C. glutamicum *IB2103 are presented. The respective values of differential gene expression shown above the arrows were deduced from DNA microarray hybridizations. Genes with decreased expression in *C. glutamicum *IB2103 are shown as white arrows; genes without a given value were detected only by real-time RT-PCR (Table 1). Predicted Rho-independent transcriptional terminators are indicated by hairpin structures, DtxR binding sites by white boxes. The DtxR binding site in front of *cg0466 *and *cg3119 *might also be involved in regulation of expression of genes on the opposite DNA strand [14]. The identified genes were grouped into seven functional classes (A-G) according to proposed physiological functions of the encoded proteins.

The 31 genes, which were characterized by differential expression pattern in the *dtxR *mutant *C. glutamicum *IB2103 and by the presence of a DtxR binding site in front of the coding region, only represent a part of the DtxR regulon since operon structures also have to be considered. Therefore, the genetic organization of the respective gene regions was analyzed by using the annotation of the *C. glutamicum *genome sequence as visualized by the GenDB database system [6]. This genome annotation contains bioinformatics predictions of Rho-independent transcriptional terminators that were generated by the sofware tool TransTerm [26]. An inspection of the genome sequence for the presence of transcriptional terminators in the downstream regions of genes that are regulated by DtxR finally resulted in the identification of twelve gene clusters or operons (Figure 5). Taking these bioinformatics predictions into account along with the functional annotation of the differentially expressed genes, at least 64 genes appeared to be directly regulated by the DtxR protein. The respective genes and gene clusters of the *C. glutamicum *genome as well as DNA microarray hybridization data regarding the differential expression in the *dtxR *mutant *C. glutamicum *IB2103 are shown in Figure 5.

The genes belonging to the DtxR regulon of *C. glutamicum *can be divided into seven functional categories (Figure 5) according to the predicted physiological roles of the deduced proteins [6]. The functional classification yielded a prominent group of 26 genes that encode iron transport systems or siderophore interacting proteins. Accordingly, this functional category includes almost half of the genes that are under direct transcriptional control by DtxR. Additionally, the genes *cg2445 *(*hmuO*), *cg2782 *(*ftn*) and *cg3327 *(*dps*) were identified as part of the DtxR regulon of *C. glutamicum*. The respective proteins specify the heme oxygenase HmuO involved in the release of iron from the heme moiety [27], a putative ferritin most likely involved in iron storage [13] and a Dps homolog that can act either in iron storage or as DNA-protecting anti-redox agent [13]. Expression of the *ftn *and *dps *genes was decreased in the *dtxR *mutant *C. glutamicum *IB2103 as was the expression of the *sdh *gene cluster that encodes the components of the iron-containing succinate dehydrogenase complex of *C. glutamicum *[6]. The genes belonging to other categories of the DtxR regulon encode proteins without a precise functional prediction, secreted proteins of unknown function or even hypothetical proteins (Figure 5), with the exception of the *cgl *gene cluster that represents the main DNA restriction and modification system of *C. glutamicum *ATCC 13032 [28] and the *cysI*-*fpr2 *gene region involved in assimilatory sulphate reduction [29].

Among the set of regulatory genes that are under direct control by DtxR, three DNA-binding transcriptional regulators were identified. This set includes the *cg1120 *(*ripA*) gene that was recently shown to be involved in iron metabolism of *C. glutamicum *[23] as well as the *cg0527 *and *cg3082 *genes encoding members of the ArsR family of regulatory proteins that typically exert metal-dependent transcriptional regulations in bacterial cells [7]. These data clearly indicate that the DtxR protein not only regulates the expression of genes playing a direct role in iron metabolism of *C. glutamicum *but also that of genes encoding transcriptional regulators, which can direct the expression of further target genes at another level of regulatory hierarchy.

### Genome-wide identification of putative DtxR binding sites in corynebacterial genome sequences and comparative content analysis of the deduced DtxR regulons

Since genes encoding a DtxR homolog are highly conserved in corynebacteria [30], the deduced genetic and functional composition of the DtxR regulon of *C. glutamicum *was compared with DtxR regulons that were predicted by bioinformatics analysis of the genome sequences of *C. efficiens *[8], *C. diphtheriae *[9] and *C. jeikeium *[10]. A multiple nucleotide sequence alignment of the 31 verified 19-bp motifs of *C. glutamicum *provided the seed information to create a HMM profile that was used to search for the presence of DtxR binding sites in the corynebacterial genome sequences. The resulting hits were applied to generate species-specific HMMs, and the genome sequences were searched again for the presence of potential DtxR binding sites with the respective profiles. This iterative approach resulted in the prediction of 15 DtxR binding sites in the genome sequence of *C. efficiens*, whereas 27 DtxR binding sites were detected in the genome sequence of *C. diphtheriae *and 21 in that of *C. jeikeium *(Table 2). With respect to the genome annotations and the bioinformatics prediction of gene clusters at least 27 genes might be under direct transcriptional control by DtxR in *C. efficiens*, 59 in *C. diphtheriae *and 51 in *C. jeikeium *(Table 2).

**Table 2:** Predicted DtxR binding sites in corynebacterial genome sequences

CDS	Predicted DtxR binding site	Clustered with	Predicted function	Orthologs
*Corynebacterium diphtheriae*				

DIP0108^1^	TTTTCTTTGCCTAGCCTAA	DIP0109–0110	iron ABC transport system	JK0661
DIP0124^1^	TTAGGGAACTCTTGCCTTA	-	membrane protein	-
DIP0169	TTAGCTTAGCCCTAGCTAA	-	secreted protein	-
DIP0222^1^	TTAGGATAGCTTTACCTAA	-	diphtheria toxin precursor	-
DIP0370	TTAGGTCAGGGTACCCTAA	DIP0371–0373	succinate dehydrogenase complex	*cg0445*, CE0386
DIP0415	TTAGCTTAACCTTGCCTAT	-	ArsR-family regulatory protein	*cg0527*, CE0466
DIP0540^1^	CTAGGTTAGGGGTGCCTAA	DIP0541	preprotein translocase subunit SecY	-
DIP0579	TTAGGCGACGGTTGCCTCA	-	hypothetical protein	-
DIP0582^1^	TTAGGGTTGTGTTACCTTG	DIP0583–0585	iron ABC transport system	-
DIP0586^1^	TTAGGGTAGCTTCGCCTAA	DIP0587–0588	siderophore biosynthesis protein	-
DIP0625^1^	TTAGGTAAGTGTAGCCTAT	DIP0624–0629^3^	iron ABC transport system	*cg0466*, JK0315
DIP0699	TTGTGTTAGCCTAGGCTAA	-	preprotein translocase subunit SecA	-
DIP0894^1^	CTAGGATTGCCTACACTTA	-	hypothetical protein	CE1009
DIP0922^1^	TAACCTTAGGCTTGCCTTT	-	AraC-family regulatory protein	*cg1120*
DIP1061^1^	TTAGGGTAACCTGTCCAAC	DIP1062-1059^3^	iron ABC transport system	-
DIP1190	ATGGGGGAGGCTCACATAA	-	peptide transport protein	-
DIP1296^1^	TTAGGGTGGGCTAACCTGC	DIP1295-1290	DNA binding protein	JK0985
DIP1520^1^	TTAGGTTAACCTTGCTTAA	DIP1519	membrane proteins	-
DIP1626	TGATGGAAACCACCCCTAA	DIP1625-1624	ubiquinol-cytochrome C reductase	-
DIP1669^1^	TGAGGGGAACCTAACCTAA	-	heme oxygenase	*cg2445*
DIP1866	TTATGCTGGGCTATCTTAA	-	bacterioferritin-like protein	*cg2782*, CE2420
DIP2114^1^	AAAGGTAAGCCATAGCTAA	-	alcohol dehydrogenase	-
DIP2161^1^	TTGGATTAGCCTACCCTAA	DIP2158–2160	non-ribosomal peptide synthase	-
DIP2202	TGAAGGTACCCCAGCCTAA	-	choline dehydrogenase	-
DIP2219	CTGGGGAACCGTTACCTAA	DIP2220	hypothetical proteins	-
DIP2303	TAAGGATAGGCCACCCCAA	-	Dps protein	*cg3327*
DIP2330	ATAGGCATGCCTAACCTCA	-	membrane protein	-

*Corynebacterium efficiens*				

CE0125	TTAGGCTAACCTTGCCCAA	-	hypothetical protein	*cg0160*
CE0386	CGAGGTGAGGCTAGCCTAA	CE0387–0389	succinate dehydrogenase complex	*cg0445*, DIP0370
CE0466	ATAGCTTAGGCTTACCTGC	-	ArsR-family regulatory protein	*cg0527*, DIP0415
CE0687	GTTGGACACCCTAACCTAA	CE0683–0686	iron ABC transport system	*cg0771*
CE0881	TTAGGTACCCTAACCTCAC	CE0882–0884	iron ABC transport system	*cg0926*, JK1887
CE0912	GTAGGTTACGCGAACGTAG	-	hypothetical protein	*cg0955*
CE1009	TTAGGCATCCCTTGCCTCG	CE1010	hypothetical proteins	DIP0894
CE1346	AGAGTGTAGGCTTACCTAT	-	hypothetical protein	*cg1405*
CE1860	TTAGGTTATAGTTTCCTTT	CE1859	hypothetical proteins	-
CE1917	GTGGGTGAGGCAAGCCTAA	-	phage integrase/recombinase	-
CE1940	TGAAGTAACACTACCCTAA	-	cation transporting P-type ATPase	-
CE2420	TTATGGTGCGCTAACCTTG	-	ferritin-like protein	*cg2782*, DIP1866
CE2790	TCAGGAAAGGTTAGCCCAA	-	hypothetical protein	-
CE2815	CTATGTTTGGCAAGCCTTA	-	hypothetical protein	-
CE2891	CTGGGCTAGGGTCACCTAT	-	hypothetical protein	-

*Corynebacterium jeikeium*				

JK0030	TTCTTTCAGGCTAACCTAT	-	DNA binding protein	-
JK0314	TTAGGTAAGGCTCGACTTA	-	membrane protein	-
JK0315	TAAGGTAACACTATCCTAA	JK0316–0319	iron ABC transport system	*cg0466*, DIP0625
JK0434	TTAGGTAAGCCTTACCTTT	JK0435–0438	ABC transport system	-
JK0461	TTAGGTTTGGCTTGGCGAA	-	collagen binding protein	-
JK0561	ACTGGCAAGGCTAAGCTAA	JK0560-0559	iron ABC transport system	DIP0108
JK0985	TTTTCTTGGTCTAACCTAA	JK0984-0979	Fe-S cluster assembly system	DIP1296
JK1470	TACGTTTTGCGTAACCTCA	-	secreted protein	-
JK1778	TTAGCTTAGGTTTACCTAT	-	enterochelin esterase	-
JK1780	ATAGGTTAGCCTACCCTTT	-	L-ornithine N_5_-oxygenase	-
JK1783	TTAGGTTATGCTAAGTTAA	JK1782-1781	ABC transporter	-
JK1805	TTAGGCAAGGGTAAGCTAA	-	iron utilization protein	-
JK1809	ACAGGTTAGGCTAACCAGA	JK1808-1806	ABC transport system	-
JK1812	TTAGGTAAGGCTACCATCA	-	solute binding protein	-
JK1815	CAAGTGAAGGCTTACCTTA	JK1816–1818	iron ABC transport system	-
JK1819	CTTGATTAGCCTAACCTAA	JK1820–1821	siderophore synthesis system	-
JK1887^2^	TTAAGCAAGGCTTGACTAA	JK1886-1884	iron ABC transport system	*cg0926*, CE0881
	TAAGGTTCGGCTAACTGTA			
JK1934	GCAGGTGACGCTAACCTGT	-	HTH_3-family regulatory protein	-
JK1979	GTAGCCTAGCCTTACCTAA	-	ornithine cyclodeaminase	-
JK1983	GAAGGTGTGGCTAACCTAA	JK1984–1985	iron ABC transport system	-

^1 ^*C. diphtheriae *genes described previously as part of the DtxR regulon [14, 15]

^2 ^Two DtxR binding sites were identified in front of the coding region.

^3 ^A single DtxR binding site is involved most likely in regulation of adjacent coding regions located on the opposite DNA strand [14, 15].

**Figure 6:**
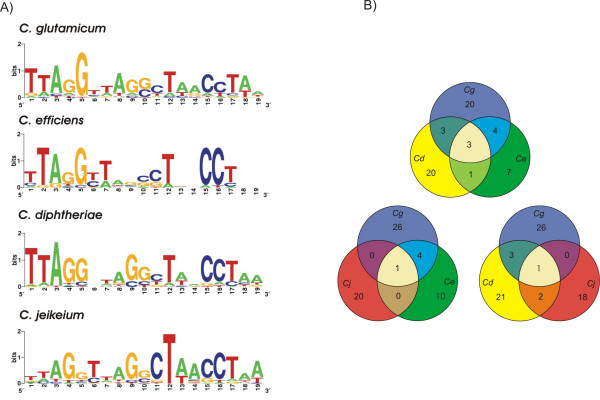
Comparative analysis of the DtxR regulons of sequenced corynebacterial species. (A) Deduced consensus sequences of DtxR binding sites represented by sequence logos. (B) Comparative content analysis of DtxR binding sites belonging to the DtxR regulon. The Venn diagrams show the number of shared and species-specific DtxR binding sites among the genomes of *C. glutamicum *(*Cg*), *C. efficiens *(*Ce*), *C. diphtheriae *(*Cd*), and *C. jeikeium *(*Cj*).

Moreover, the identified 19-bp motifs were used to generate species-specific consensus sequences of the DtxR binding sites by means of the WebLogo tool [31]. The resulting sequence logos are shown in Figure 6A. The overall height of each stack of letters indicates the sequence conservation at each position of the 19-bp motif, wheras the heigth of each symbol within the stack reflects the relative frequency of the corresponding nucleotide at that position. A comparison of the sequence logos clearly indicated that the four 19-bp motifs resemble the overall palindromic consensus sequence 5'-TTAGGTTAG(G/C)CTAACCTAA-3' of DtxR binding sites, with apparent species-specific variations (Figure 6A). Accordingly, the sequenced corynebacterial genomes not only encode a conserved DtxR homolog but also contain sets of similar 19-bp motifs that represent potential DtxR binding sites. These data along with the verified DtxR binding sites of *C. glutamicum *and the annotated genome sequences of *C. efficiens*, *C. diphtheriae *and *C. jeikeium *enabled us to analyze and compare the genetic composition of the DtxR regulons in these corynebacterial species.

To identify either species-specific or common genes that are under transcriptional control by DtxR in corynebacteria, functional predictions of the coding regions located downstream of the determined DtxR binding sites were compared between the four sequenced species. Orthologous proteins were identified within the complete collection of potentially DtxR-regulated genes by using the BLASTP algorithm [32] to detect amino acid sequence similarities and by performing synteny analyses of the respective genomic context. The resulting data were visualized as Venn diagrams (Figure 6B). First of all, it is apparent that the four corynebacteria share no common gene that is under direct transcriptional control by the DtxR regulator. This unexpected result might be due to the exceptional collection of species-specific genes that are involved in iron metabolism of *C. jeikeium *[10]. The set of orthologous genes that are most likely under transcriptional regulation by DtxR in three corynebacterial species includes the orthologs of the *cg0466 *gene cluster (Figure 5) that encode components of an iron ABC transport system in *C. glutamicum*, *C. diphtheriae *and *C. jeikeium *and the orthologs of the *cg0926 *gene cluster that are apparently involved in iron uptake in *C. glutamicum*, *C. efficiens *and *C. jeikeium*. Orthologs of the *sdh *gene cluster and the *ftn *gene, whose expression decreased in the *dtxR *mutant *C. glutamicum *IB2103, are apparently under direct transcriptional control by DtxR in *C. efficiens *and *C. diphtheriae*, indicating that the DtxR regulator might be involved in the activation of gene expression also in these species. Regulatory genes orthologous with *cg0527 *of *C. glutamicum *are present in the genomes of *C. efficiens *and *C. diphtheriae *[7] and all are presumably regulated by DtxR, suggesting similar hierarchical topologies of the respective DtxR regulons. However, most of the identified genes that are under transcriptional control by DtxR are species-specific, although one has to keep in mind that some non-orthologous genes might share similar physiological functions. This comparative content analysis led to the conclusion that the DtxR regulons of corynebacteria are quite different in their genetic composition, although they rely on an orthologous regulatory protein and similar 19-bp motifs for regulator binding. Nevertheless, some interesting regulatory features deduced from the experimental characterization of the DtxR regulon of *C. glutamicum *are apparently realized also in the topology of DtxR regulons of other corynebacteria.

## Discussion

In the present study, the genetic network of the transcriptional regulator DtxR was examined in *C. glutamicum*, and the topology of the DtxR regulon was deduced from DNA microarray hybridizations, bioinformatics approaches, *in vivo *studies of differential gene expression, and DNA band shift assays. For these purposes, a defined deletion was constructed within the coding region of the *dtxR *gene, resulting in the mutant strain *C. glutamicum *IB2103 that was unable to grow in CGXII minimal medium under high-iron conditions. However, when the *dtxR *mutant *C. glutamicum *IB2103 was cultivated in low-iron CGXII medium, it revealed the same growth behavior as the wild-type control. These experiments showed that the *dtxR *gene is dispensable in *C. glutamicum *under specific culture conditions, as it was previously demonstrated for the orthologous regulatory genes *dtxR*, *dmdR1 *and *ideR *of *C. diphtheriae*, *Streptomyces coelicolor *and *Mycobacterium smegmatis*, respectively [33-35]. On the other hand, the *ideR *gene of *Mycobacterium tuberculosis *can be inactivated only in the presence of a second functional copy of the gene or when a second-site suppressor mutation alleviates the lethal effects of *ideR *inactivation, making *ideR *an essential gene in this bacterial species [36].

As observed with the defined *dtxR *mutant strain *C. glutamicum *IB2103, growth of the *dtxR *transposon mutant C7(β)18.5 of *C. diphtheriae *was dependent on the amount of iron that was added to the culture medium [33]. The C7(β)18.5 mutant of *C. diphtheriae *exhibited an extended lag phase and slower exponential growth in low-iron medium when compared with the parental C7(β) wild-type strain. Moreover, the C7(β)18.5 mutant grew only poorly in high-iron medium when compared with a control medium containing no additional iron sources, and it was more-easily killed by exposure to high-iron conditions and H_2_O_2 _than was the parental strain [33]. Since the DtxR protein apparently regulates the expression of iron uptake systems, it might also be involved in protecting the cell from damage that is caused by high intracellular iron concentrations. Transcriptional deregulation of iron homeostasis favors the Fenton reaction, leading to the production of hydroxyl radicals that can damage all biological macromolecules [37]. Therefore, transcriptional regulation of iron metabolism by the DtxR protein in *C. glutamicum *is not only necessary for economic reasons but also to avoid inevitable iron toxicity. In *E. coli*, iron homeostasis and oxidative stress response are closely interconnected and strictly controlled by the transcriptional regulator Fur [38]. Inactivation of the *fur *gene enhanced the sensitivity of the cell to redox stress, a physiological effect that can be reversed by iron chelation, by inhibiting ferric iron transport or by enhancing the iron storage capacity of the cell [37].

The *dtxR *mutant *C. glutamicum *IB2103 was further on used to compare the genome-wide expression pattern with that of the wild-type strain by DNA microarray hybridizations. In principle, inactivation of a gene encoding a repressor results in constitutive expression of those genes that are directly regulated by the respective protein. Therefore, comparative DNA microarray hybridizations are typically carried out in such a way that the relevant genes of the wild-type control are repressed and those of the mutant strain are de-repressed [39]. Since the *dtxR *mutant *C. glutamicum *IB2103 grew only in low-iron medium containing a trace amout of iron, the genes of the DtxR regulon are apparently de-repressed in both the mutant strain and the wild-type control. To detect differential gene expression in *C. glutamicum *IB2103, we applied an alternative experimental setup by the subsequent addition of an appropriate amount of iron to low-iron growth medium. Indeed, the DtxR repressor switched off the expression of its target genes in the wild-type strain, whereas the target genes were further on expressed in the *dtxR *mutant. This physiological effect was detectable on a global scale by DNA microarray experiments and enabled the screening for differentially expressed genes that were later on assigned to the DtxR regulon of *C. glutamicum*. In addition to genes belonging to the DtxR regulon, the DNA microarray hybridization identified numerous coding regions whose expression was positively or negatively influenced in response to the iron stimulus. These genes are considered to be subject to indirect regulation by iron and are apparently not part of the DtxR regulon of *C. glutamicum *since they lack DtxR binding sites in their upstream regions. Investigating the physiological role of these genes in the cellular response to the subsequent addition of iron might eventually allow a better understanding of the molecular mechanisms used by *C. glutamicum *to counter high-iron conditions.

Furthermore, the combination of bioinformatics predictions and DNA band shift assays resulted in the identification of DtxR binding sites within the upstream region of 31 genes and gene clusters, suggesting that at least 64 genes are under direct transcriptional control by DtxR in *C. glutamicum*. For a closer examination of the identified DtxR binding sites, transcriptional start sites of four differentially expressed genes were determined and the corresponding promoters were mapped. Three DtxR binding sites were found to overlap the deduced promoter regions, suggesting that DtxR acts as transcriptional repressor by blocking RNA polymerase binding and thus preventing expression of the respective genes [25]. Similar results were reported upon promoter mapping and localization of DtxR binding sites in *C. diphtheriae *[40] and of IdeR binding sites in *M. smegmatis *and *M. tuberculosis *[41,42]. On the other hand, the DtxR binding sites of *cg2782*, *cg3327 *and *cg0445 *were located upstream of the deduced -35 promoter region. Since the respective genes showed a decreased expression in *C. glutamicum *IB2103 during DNA microarray hybridization, this location is more consistent with an activating function of the corresponding transcriptional regulator [25]. In general, transcriptional regulators activate the expression of genes in such a way that they facilitate interaction of the RNA polymerase with the respective promoters. However, the large distance of the DtxR binding site to the -35 promoter region of the *cg0445 *gene cluster that encodes the iron-containing succinate dehydrogenase complex of *C. glutamicum *suggests an alternative activation mechanism. In this context it is noteworthy that the *cg0445 *gene cluster is also under direct transcriptional control by the RipA repressor [23], a member of the AraC-family of DNA-binding transcriptional regulators [7]. Some members of the AraC protein family fulfill their regulatory function by using two DNA binding sites and a DNA looping mechanism to control gene expression [43]. The DtxR binding site in front of *cg0445 *is located directly adjacent to the RipA binding site A, and DtxR binding might therefore interfere with RipA binding to exert its regulatory function. Since expression of the *ripA *gene is also controlled by DtxR, the corresponding network topology of the DtxR regulon represents a coherent feed-forward loop of type 4, in which the direct connection to the target gene is activating and the indirect connections are both repressing [44]. In such a way environmental input signals can be integrated into the transcriptional regulatory network at different levels of regulatory hierarchy to control expression of the succinate dehydrogenase complex.

In addition to the *cg0445 *gene cluster, the *dps *(*cg3327*) and *ftn *(*cg2782*) genes showed a decreased expression in the *dtxR *mutant *C. glutamicum *IB2103. Dps-like proteins may function either as anti-redox agents or iron storage proteins, whereas ferritins act primarily in iron storage [13]. These data indicated that the DtxR protein can exert a regulatory role as positive modulator of expression of genes that are involved in iron storage and DNA protection. This mechanism of transcriptional regulation is reasonable since the iron storage and protection genes are not expressed under low-iron conditions, whereas both, the iron storage capacity of the cell and the protection of DNA, increase under high-iron conditions by activating the respective genes. In *M. tuberculosis*, expression of the iron storage genes *bfrA *and *bfrB*, encoding a bacterioferritin and a ferritin-like protein, is induced also by iron and IdeR [36,42]. Binding of IdeR to the regulatory region of *bfrA *indicated that the gene is transcribed from a promoter that is activated by iron and binding of IdeR, since induction of *bfrA *gene expression was detected in the wild-type but not in an *ideR *mutant strain. The transcriptional control of iron storage in *E. coli *was also shown to be positively regulated by iron but no direct interaction of Fur with the regulatory region of the *bfrA *and *ftn *genes has been observed [45,46].

The comparative content analysis of DtxR binding sites in four corynebacterial genome sequences provided further insights into the topology of the DtxR regulon of *C. glutamicum*. The deduced consensus sequences of DtxR binding sites are not only very similar among the corynebacterial species but also resemble IdeR and DmdR1 binding sites that were detected in *M. tuberculosis *and *S. coelicolor*, respectively [42,47]. Consequently, DtxR-like regulatory proteins utilize conserved recognition signals in different actinobacteria, as is the situation with DNA binding sites of the Fur repressor in gamma-proteobacteria [38]. On the other hand, the comparative content analysis revealed that most of the DtxR-regulated genes are species-specific in the four sequenced corynebacteria, indicating that a wide variety of genetic information is used in the individual species to provide an effective iron homeostasis. Varying genetic information can be gained for instance by horizontal gene transfer, as suggested for the *C. jeikeium *genome that contains large sets of DtxR-regulated iron acquisition genes on mobile genetic elements [10]. As iron metabolism is closely connected to iron toxicity, any additional genetic information regarding iron metabolism has to be integrated into the pre-existing DtxR regulon to avoid detrimental consequences for the bacterial cell. Only in case of similar DNA binding sites being present in the regulatory region of the respective genes, it is generally ensured that the genes fall under direct regulation by the DtxR protein and that the bacterium can benefit from the acquired genetic information. The close functional relationship between DNA binding sites of at least DtxR and IdeR is indeed apparent from the observation that the regulatory proteins can interact with operators of the cognate regulons in either corynebacteria or mycobacteria [48].

## Conclusion

In summary, the presented experimental work adds considerably to our currrent understanding of the transcriptional regulatory network of *C. glutamicum *genes that are controlled by the diphtheria toxin repressor homolog DtxR and of the response of this bacterium to changing environmental iron levels. The results demonstrate that DtxR acts as a dual transcriptional regulator with a major role in controlling the expression of genes involved in iron metabolism. The DtxR protein exerts its dual regulatory function as repressor of genes participating in iron uptake and iron utilization and as activator of genes responsible for iron storage and DNA protection. Moreover, the data suggest that the DtxR protein acts as global regulator by controlling the expression of other regulatory proteins that might take care of an iron-dependent regulation of a broader transcriptional network of *C. glutamicum *genes.

## Methods

### Bacterial strains and growth conditions

Wild-type strain *C. glutamicum *ATCC 13032 (American Type Culture Collection, Manassas, VA) was used to characterize the DtxR regulon. *C. glutamicum *strains were routinely grown at 30°C in Luria-Bertani medium [49]. Growth of *C. glutamicum *strains in CGXII minimal medium [50] containing 30 mg/l protocatechuic acid was monitored in time intervals of 1 h with the nephelometer Nephelostar Galaxy (BMG Laboratories, Offenburg, Germany). Standard CGXII minimal medium (10 mg/l FeSO_4_) and low-iron CGXII medium (0 mg/l FeSO_4_) were used for growth assays [33]. Growth of shaking flask cultures was monitored by measuring the optical density at 600 nm with an Eppendorf *Bio*Photometer. *E. coli *DH5αMCR was used for standard cloning procedures and grown at 37°C in Luria-Bertani medium [49] supplemented with 2 g/l glucose. Selection for the presence of plasmids was performed with kanamycin (50 μg/ml for *E. coli *and 25 μg/ml for *C. glutamicum*). Isopropyl beta-D-thiogalactoside (IPTG) was used to induce *dtxR *expression by the *P*_*trc *_promoter of pEC-XK99E [22].

### DNA isolation, manipulation and transfer

Vector DNA was prepared from *E. coli *cells by an alkaline lysis technique using the QIAprep Spin Miniprep Kit (Qiagen, Hilden, Germany). Chromosomal DNA of *C. glutamicum *was prepared as described previously [51]. Modification of DNA, analysis by agarose gel electrophoresis and ligation were performed by standard procedures [49]. Transformation of *E. coli *and *C. glutamicum *cells was performed by electroporation [52,53].

### PCR techniques and dtxR mutant construction

PCR experiments were carried out with a PTC-100 thermocycler (MJ Research, Watertown, MA), Pwo DNA polymerase (Roche Diagnostics, Mannheim, Germany) and chromosomal *C. glutamicum *DNA as template. PCR products were purified by using the PCR Purification Spin Kit (Qiagen). Oligonucleotides used for PCR amplification were purchased from Operon Biotechnologies (Cologne, Germany). The gene SOEing method [54] was applied to construct a pK18*mobsacB *derivative that is suitable to perform an allelic exchange of the *dtxR *gene in the chromosome of *C. glutamicum *ATCC 13032 [19]. The primers used were cg2103del1 (GATCTAGAATTCCCAAGGCGTGAGATGACAG), cg2103del2 (GAGCACGCAGAGGAACAAT), cg2103del3 (ATTGTTCCTCTGCGTGCTCTCAAGCAGATGAGCCTGAT), and cg2103del4 (GATCTAGGATCCTTCTACGCGGACTGCATGT). The DNA fragment was digested with EcoRI and BamHI and cloned into the vector pK18*mobsacB*. The resulting plasmid pIB2103 carries a modified *dtxR *gene that is specified by a defined deletion of 338 nucleotides. Gene replacement in the chromosome of *C. glutamicum *ATCC 13032 resulted in the *dtxR *mutant strain *C. glutamicum *IB2103. For genetic complementation of mutant strain *C. glutamicum *IB2103, the *dtxR *coding region was amplified by PCR with the primer pair compl1 (GATCTAGAATTCAAAGGAGGACAACCATGAAGGATCTGGTCGATAC) and compl2 (GATCTAGGATCCGTGTGTTAGCCCTCAACC). The PCR product was digested with EcoRI and BamH and cloned into the compatible sites of the IPTG-inducible expression vector pEC-XK99E [22]. The resulting plasmid pIB4000 was transferred into *C. glutamicum *ATCC 13032 and *C. glutamicum *IB2103 by electrotransformation.

### Total RNA preparation and DNA microarray hybridization

For the preparation of total RNA, *C. glutamicum *cultures were grown in minimal medium CGXII. Approximately 1 × 10^9 ^cells from exponentially growing cultures were harvested by centrifugation with 11,000 *g *for 15 s and subsequently transferred into liquid nitrogen. RNA isolation, cDNA synthesis, labeling of probes, and DNA microarray hybridization were performed as described previously [20]. The experiments were carried out in duplicate using label swapping. Normalization and evaluation of data was accomplished by the EMMA microarray data analysis software using a *m*-value cut-off of ± 1, which corresponds to relative expression changes equal or greater than twofold [20]. Since each DNA microarray contains four replicates per gene, a total number of eight spots per gene was available for calculating differential gene expression.

### Bioinformatics tools for DtxR binding site prediction

The annotated version of the *C. glutamicum *ATCC 13032 genome sequence [6] was used to perform a genome-wide screening for putative DtxR binding sites. The search was accomplished by Hidden Markov model (HMM) analysis using the HMMER software package [55]. A set of predicted DtxR binding sites was aligned by means of the CLUSTAL X program [21], and the resulting alignment was used to create a HMM by using the HMMBUILD module. The calculated HMM profile along with the HMMSEARCH module was applied to screen the *C. glutamicum *genome sequence for the presence of DtxR binding sites. The genomic positions of the resulting hits were correlated with coding sequences that revealed differential expression in the *dtxR *mutant when compared with the wild-type strain by DNA microarray hybridization. The experimentally verified DtxR binding sites of *C. glutamicum *were aligned with the CLUSTAL X program and used to generate a HMM profile for an iterative screening of the genome sequences of *C. efficiens *[8], *C. diphtheriae *[9] and *C. jeikeium *[10].

### Real-time reverse transcription (RT)-PCR

Purified total RNA of *C. glutamicum *cultures was used for real-time RT-PCR experiments with the LightCycler instrument (Roche Diagnostics) and the Quanti-Tect SYBR Green RT-PCR Kit (Qiagen). Oligonucleotides used to measure relative gene expression were purchased from Operon Biotechnologies. Verification of RT-PCR products was performed by melting curve analysis. Differences in gene expression were determined by comparing the crossing points of two samples measured in duplicate. Crossing points were calculated by the LightCycler software (Roche Diagnostics).

### Construction and purification of a His-tagged DtxR protein

A PCR product fusing the coding region of the *C. glutamicum dtxR *gene with a nucleotide sequence encoding a C-terminal His tag was generated by using the primer pair dtxR-His1 (GATCTAGAATTCAAAGGAGGACAACCATGAAGGATCTGGTCGATA) and dtxR-His2 (GATCTAGGATCCTTAATGGTGATGGTGATGGTGGCCCTCAACCTTTTCTAC). The PCR product was digested with EcoRI and BamHI and cloned into the IPTG-inducible expression vector pEC-XK99E [22]. The resulting plasmid pIB4001 was transferred to *E. coli *DH5αMCR and to *C. glutamicum *strains by electroporation. To isolate the His-tagged DtxR protein, *E. coli *DH5αMCR (pIB4001) was grown for 16 h in Luria-Bertani medium containing 50 μg/ml kanamycin and 0.1 mM IPTG. Approximately 4 × 10^10 ^cells were harvested by centrifugation, resuspended in LEW buffer (50 mM NaH_2_PO_4_, 300 mM NaCl, one tablet of Roche Complete Mini protease inhibitors; pH 8.0) and transferred into a RiboLyser tube (Hybaid, Heidelberg, Germany). Cell disruption by means of the RiboLyser instrument was carried out with a speed rate of 6.5 and two time intervals of 30 s. The His-tagged DtxR protein was purified from the protein crude extract with Protino Ni-TED 1000 packed columns (Macherey-Nagel, Düren, Germany) according to the manufacturer's instructions. After loading of the protein crude extract, the resin column was washed twice with 2 ml LEW buffer. The His-tagged DtxR protein was eluted with 2.5 ml LEW buffer containing 10 mM imidazole and finally stored at -20°C. The resulting eluate was concentrated by using Amicon ultra-4 5000 MWCO centrifugal filter units (Millipore, Schwalbach, Germany) and subsequently analyzed by SDS-PAGE [53]. The protein concentration was determined with the Bio-Rad protein assay kit (Bio-Rad Laboratories, Munich, Germany). To verify the purification of the His-tagged DtxR protein, an aliqout of the eluate was enzymatically digested with modified trypsin (Promega, Mannheim, Germany) and the resulting peptide mass fingerprint was determined by MALDI-TOF mass spectrometry, applying an Ultraflex mass spectrometer (Bruker Daltonics, Bremen, Germany) and the MASCOT software.

### DNA band shift assays to characterize binding of the purified DtxR protein

DNA band shift assays were performed with Cy3-labeled 40 mer oligonucleotides (Operon) that were annealed with corresponding complementary oligonucleotides to double-stranded DNA fragments by heating at 94°C for 5 min and annealing on ice for 15 min. During band shift assays, 42 pmol of purified His-tagged DtxR protein were mixed with 0.05 pmol DNA, 200 μM CoCl_2_, 15% (v/v) glycerol, and DtxR binding buffer (20 mM Na_2_HPO_4_, 50 mM NaCl, 5 mM MgCl_2_, 2 nM DTT, 10% (v/v) glycerol, 100 μg/ml BSA; pH 7.0 [56]), to get a total volume of 20 μl. The assay was incubated at room temperature for 15 min and then separated with a 2% agarose gel (containing 150 mM CoCl_2_) prepared in gel buffer (20 mM Na_2_HPO_4_; pH 7.0). A voltage of 80 V was supplied for 1.5 h. The agarose gel was scanned with a Typhoon 8600 Variable Mode Imager (Amersham Biosciences Europe, Freiburg, Germany). During displacement experiments, increasing concentrations of non-labeled competitor DNA (0.05 to 1 pmol) were added to the reaction batch.

### Identification of transcriptional start sites by the RACE method

For the identification of transcriptional start sites, total RNA was isolated from *C. glutamicum *wild-type cultures grown in CGXII medium. RACE primers (18 mer oligonucleotides) binding 200 to 300 nucleotides downstream of the annotated translational starts of investigated genes along with 1 μg of total RNA were used for cDNA synthesis. RACE primer sequences will be provided by the authors upon request. The resulting cDNA was modified and amplified by two additional PCRs using the 5'/3' RACE Kit second generation (Roche Diagnostics). PCR products were cloned into the pCR2.1-TOPO vector (Invitrogen, Karlsruhe, Germany) and transferred into competent *E. coli *TOP10 cells. DNA sequencing of cloned RACE products was performed by IIT Biotech (Bielefeld, Germany).

## Authors' contributions

IB carried out the experimental work and drafted the manuscript. HW participated during real-time RT-PCR and RACE PCR experiments. ATH provided the DNA microarray. JK participated in data evaluation. AP conceived of the design of the figures and participated in supervision. AT conceived of the study and participated in co-ordination and supervision. All authors read and approved the final manuscript.

## Supplementary Material

Additional file 1 *C. glutamicum *genes transcriptionally up-regulated or down-regulated in the *dtxR *mutant IB2103 upon iron addition to the growth medium when compared to the wild-type ATCC 13032. Relevant molecular and expression data of all genes detected as differentially expressed in *C. glutamicum *IB2103 upon addition of iron to the growth medium Click here for file
