# Supplementary material for: The DtxR protein acting as dual transcriptional regulator directs a global regulatory network involved in iron metabolism of Corynebacterium glutamicum
Source: BMC Genomics. 2006 Feb 9;7:21. doi: 10.1186/1471-2164-7-21 (PMC1382209; doi:10.1186/1471-2164-7-21)
Supplement: Additional file 1 — C. glutamicum genes transcriptionally up-regulated or down-regulated in the dtxR mutant IB2103 upon iron addition to the growth medium when compared to the wild-type ATCC 13032. Relevant molecular and expression data of all genes detected as differentially expressed in C. glutamicum IB2103 upon addition of iron to the growth medium [file 1471-2164-7-21-S1.pdf]

**Additional file 1: *C. glutamicum* genes transcriptionally up-regulated or down-regulated in the *dtxR* mutant IB2103 upon iron addition to the growth medium when compared to the wild-type ATCC 13032**

| CDS[1] | Gene | Gene product or deduced function | COG class | *M*-value | *A*-value | *p*-value |
|---|---|---|---|---|---|---|
| Genes with enhanced expression in IB2103 | | | | | | |
| *cg2445** | *hmuO* | putative heme oxygenase | P | 5.60 | 12.55 | $8.95 \times 10^{-14}$ |
| *cg1931** | - | putative secreted protein | X | 5.57 | 11.76 | $1.13 \times 10^{-09}$ |
| *cg2797** | - | conserved hypothetical protein | S | 5.39 | 11.46 | $7.24 \times 10^{-09}$ |
| *cg2796** | - | MMGE/PRPD family protein | R | 5.38 | 12.19 | $1.91 \times 10^{-09}$ |
| *cg0467** | - | cobalamin/Fe3+-siderophores transport system | P | 5.23 | 11.38 | $7.54 \times 10^{-11}$ |
| *cg1903* | - | ABC-type multidrug transport system | V | 5.21 | 12.66 | $3.69 \times 10^{-10}$ |
| *cg0771** | *irp1* | DtxR/iron-regulated lipoprotein precursor | P | 5.14 | 11.67 | $2.58 \times 10^{-08}$ |
| *cg3156** | - | putative secreted protein | X | 5.06 | 11.01 | $1.08 \times 10^{-08}$ |
| *cg1120** | *ripA* | AraC-family transcriptional regulator | K | 4.99 | 12.35 | $9.15 \times 10^{-11}$ |
| *cg1930** | - | putative secreted hydrolase | O | 4.90 | 9.83 | $2.52 \times 10^{-07}$ |
| *cg0922** | - | ABC-type cobalamin/Fe3+-siderophores transport system | P | 4.50 | 10.96 | $5.85 \times 10^{-12}$ |
| *cg0924** | - | ABC-type cobalamin/Fe3+-siderophores transport system | P | 4.40 | 13.69 | $5.92 \times 10^{-09}$ |
| *cg1405** | - | siderophore-interacting protein | P | 4.36 | 13.40 | $1.11 \times 10^{-08}$ |
| *cg0921** | - | siderophore-interacting protein | P | 4.24 | 10.75 | $2.13 \times 10^{-11}$ |
| *cg0928** | - | ABC-type cobalamin/Fe3+-siderophores transport system | P | 4.22 | 11.50 | $2.11 \times 10^{-09}$ |
| *cg0926** | - | ABC-type cobalamin/Fe3+-siderophores transport system | P | 4.00 | 11.83 | $7.73 \times 10^{-07}$ |
| *cg0963* | - | hypothetical protein | X | 3.94 | 10.36 | $1.50 \times 10^{-06}$ |
| *cg2444* | - | hypothetical protein | X | 3.87 | 12.65 | $4.05 \times 10^{-09}$ |
| *cg0469* | - | ABC-type cobalamin/Fe3+-siderophores transport system | P | 3.74 | 11.93 | $7.09 \times 10^{-09}$ |
| *cg0466** | - | conserved secreted protein | X | 3.67 | 11.77 | $8.84 \times 10^{-09}$ |
| *cg0526* | - | translation initiation inhibitor | J | 3.53 | 9.81 | $6.22 \times 10^{-08}$ |
| *cg0160** | - | hypothetical protein | X | 3.49 | 12.53 | $2.44 \times 10^{-09}$ |
| *cg0927** | - | ABC-type cobalamin/Fe3+-siderophores transport system | P | 3.47 | 12.39 | $2.20 \times 10^{-08}$ |
| *cg2962* | - | conserved hypothetical protein | R | 3.42 | 12.04 | $2.53 \times 10^{-06}$ |
| *cg1419* | - | putative Na+-dependent transporter | R | 3.33 | 10.96 | $2.85 \times 10^{-08}$ |
| *cg3404** | - | ABC-type cobalamin/Fe3+-siderophores transport system | P | 3.29 | 8.66 | $2.05 \times 10^{-07}$ |
| *cg0527** | - | ArsR-family transcriptional regulator | K | 3.22 | 11.29 | $3.19 \times 10^{-10}$ |
| *cg1883* | - | putative secreted protein | S | 3.19 | 10.92 | $3.95 \times 10^{-11}$ |
| *cg2234** | - | ABC-type cobalamin/Fe3+-siderophores transport system | P | 3.18 | 11.80 | $1.62 \times 10^{-06}$ |
| *cg0159** | - | hypothetical protein | X | 3.13 | 10.50 | $3.29 \times 10^{-05}$ |
| *cg0755* | *metY* | O-acetylhomoserine (thiol)-lyase | E | 3.09 | 12.59 | $7.47 \times 10^{-07}$ |
| *cg2311** | - | SAM-dependent methyltransferase | QR | 3.04 | 10.23 | $1.44 \times 10^{-05}$ |
| *cg0468** | - | ABC-type cobalamin/Fe3+-siderophores transport systems | P | 3.03 | 12.09 | $1.61 \times 10^{-08}$ |
| *cg0748** | - | ABC-type cobalamin/Fe3+-siderophores transport systems | P | 3.01 | 10.05 | $5.23 \times 10^{-04}$ |
| *cg2381* | - | conserved hypothetical protein | X | 2.92 | 11.42 | $3.25 \times 10^{-08}$ |
| *cg1894* | - | hypothetical protein | X | 2.91 | 8.78 | $8.16 \times 10^{-08}$ |
| *cg2113* | - | hypothetical protein | X | 2.58 | 10.90 | $6.32 \times 10^{-05}$ |
| *cg3345* | - | hypothetical protein | X | 2.53 | 10.43 | $5.62 \times 10^{-07}$ |
| *cg0754* | *metX* | homoserine O-acetyltransferase | E | 2.49 | 12.06 | $1.41 \times 10^{-09}$ |
| *cg1895* | - | putative secreted protein | X | 2.43 | 8.83 | $2.42 \times 10^{-03}$ |
| *cg1567* | - | hypothetical protein | X | 2.34 | 9.47 | $1.05 \times 10^{-06}$ |
| *cg2678* | - | ABC-type dipeptide/oligopeptide/nickel transport system | E | 2.33 | 11.36 | $8.82 \times 10^{-09}$ |
| *cg1898* | - | hypothetical protein | X | 2.32 | 13.05 | $1.77 \times 10^{-09}$ |
| *cg3119** | *fpr2* | NADPH-dependent ferredoxin-reductase | ER | 2.30 | 13.79 | $4.40 \times 10^{-05}$ |
| *cg2141* | *recA* | DNA recombination/repair protein | L | 2.28 | 12.31 | $4.88 \times 10^{-12}$ |
| *cg2687* | *metB* | cystathionine gamma-synthase | E | 2.18 | 12.70 | $3.33 \times 10^{-12}$ |
| *cg0899* | - | glutamine amidotransferase | H | 2.16 | 11.16 | $1.52 \times 10^{-09}$ |
| *cg0767** | - | siderophore-interacting protein | P | 2.12 | 11.24 | $7.79 \times 10^{-06}$ |
| *cg0769** | - | ABC-type cobalamin/Fe3+-siderophores transport system | P | 2.06 | 9.75 | $5.15 \times 10^{-06}$ |
| *cg1287* | - | conserved hypothetical protein | X | 2.06 | 12.02 | $2.61 \times 10^{-05}$ |
| *cg2336* | - | putative secreted protein | X | 2.04 | 11.87 | $1.49 \times 10^{-09}$ |
| *cg2533* | - | conserved hypothetical protein | X | 2.03 | 10.38 | $7.31 \times 10^{-08}$ |
| *cg2675* | - | ATPase component of ABC-type transport system | R | 2.01 | 11.97 | $3.86 \times 10^{-06}$ |
| *cg1806* | *metK* | S-adenosylmethionine synthetase | H | 1.97 | 9.75 | $8.12 \times 10^{-06}$ |
| *cg3118** | *cysI* | ferredoxin-sulfite reductase | P | 1.94 | 13.32 | $1.73 \times 10^{-06}$ |
| *cg0898* | - | pyridoxine biosynthesis enzyme | H | 1.93 | 11.15 | $6.03 \times 10^{-07}$ |
| *cg0839* | - | hypothetical protein | X | 1.91 | 11.28 | $8.48 \times 10^{-11}$ |
| *cg0770** | - | ABC-type cobalamin/Fe3+-siderophores transport system | P | 1.90 | 10.96 | $6.55 \times 10^{-07}$ |
| *cg0156* | - | Crp-family transcriptional regulator | KG | 1.89 | 11.63 | $3.67 \times 10^{-07}$ |
| *cg1404* | *gatA* | putative Glu-tRNA amidotransferase | J | 1.88 | 11.89 | $7.76 \times 10^{-09}$ |
| *cg1896* | - | putative secreted protein | X | 1.87 | 10.87 | $1.85 \times 10^{-06}$ |
| *cg1418** | - | ABC-type cobalamin/Fe3+-siderophores transport system | P | 1.85 | 11.89 | $1.57 \times 10^{-06}$ |

| | | | | | | |
|---|---|---|---|---|---|---|
| *cg3374* | - | putative NADH-dependent flavin oxidoreductase | C | 1.81 | 10.66 | $6.45 \times 10^{-08}$ |
| *cg1377* | *ssuC* | ABC-type aliphatic sulfonate transporter | P | 1.76 | 9.72 | $1.51 \times 10^{-08}$ |
| *cg1996** | *cglIM* | modification methylase | L | 1.72 | 11.80 | $5.35 \times 10^{-09}$ |
| *cg0768** | - | ABC-type cobalamin/Fe3+-siderophores transport system | PH | 1.65 | 10.94 | $1.21 \times 10^{-05}$ |
| *cg3049* | *fpr1* | NADPH-dependent ferredoxin-reductase | ER | 1.61 | 11.53 | $1.19 \times 10^{-08}$ |
| *cg3106* | - | conserved hypothetical protein | X | 1.55 | 9.52 | $3.31 \times 10^{-08}$ |
| *cg2443* | - | permease of the major facilitator superfamily | X | 1.54 | 11.01 | $1.53 \times 10^{-06}$ |
| *cg2950* | *radA* | putative ATP-dependent protease | O | 1.54 | 11.13 | $3.16 \times 10^{-07}$ |
| *cg0838* | - | helicase family protein | R | 1.53 | 11.12 | $3.13 \times 10^{-09}$ |
| *cg1337* | *hom* | homoserine dehydrogenase | E | 1.53 | 13.58 | $1.35 \times 10^{-10}$ |
| *cg0544* | - | putative membrane protein | X | 1.53 | 9.32 | $7.90 \times 10^{-09}$ |
| *cg1848* | - | putative reductase | C | 1.52 | 9.85 | $1.42 \times 10^{-08}$ |
| *cg3372* | - | conserved hypothetical protein | S | 1.52 | 12.83 | $7.80 \times 10^{-05}$ |
| *cg1171* | - | putative GTPase | J | 1.52 | 10.08 | $5.21 \times 10^{-03}$ |
| *cg1451* | *serA* | phosphoglycerate dehydrogenase | E | 1.49 | 14.17 | $4.03 \times 10^{-11}$ |
| *cg0545* | *pitA* | putative low-affinity phosphate transport protein | P | 1.48 | 9.68 | $8.78 \times 10^{-07}$ |
| *cg1658* | - | permease of the major facilitator superfamily | X | 1.46 | 9.69 | $2.56 \times 10^{-07}$ |
| *cg0053* | - | putative iron-siderophore uptake system | PH | 1.45 | 9.90 | $8.60 \times 10^{-05}$ |
| *cg1626* | - | conserved hypothetical protein | S | 1.44 | 10.93 | $1.25 \times 10^{-06}$ |
| *cg1356* | - | putative rRNA or tRNA methylase | J | 1.44 | 10.46 | $5.11 \times 10^{-07}$ |
| *cg2810* | - | Na+/H+-dicarboxylate symporter family | C | 1.44 | 9.32 | $2.52 \times 10^{-09}$ |
| *cg0736* | - | ABC-type transporter. ATP-binding protein | P | 1.43 | 11.73 | $4.38 \times 10^{-10}$ |
| *cg2140* | *recX* | regulatory protein involved in DNA repair | R | 1.42 | 9.80 | $4.71 \times 10^{-06}$ |
| *cg0737* | - | ABC-type transporter. substrate-binding protein | P | 1.42 | 11.79 | $5.10 \times 10^{-03}$ |
| *cg1962* | - | putative membrane protein | X | 1.42 | 10.29 | $3.54 \times 10^{-08}$ |
| *cg0435* | *udgA1* | UDP-glucose 6-dehydrogenase | M | 1.40 | 12.33 | $1.59 \times 10^{-05}$ |
| *cg1602* | *recN* | DNA repair protein RecN | L | 1.40 | 10.61 | $6.28 \times 10^{-08}$ |
| *cg0715* | - | secreted protein | X | 1.40 | 10.44 | $2.92 \times 10^{-06}$ |
| *cg2312* | *gip* | putative hydroxypyruvate isomerase protein | G | 1.38 | 8.17 | $2.30 \times 10^{-05}$ |
| *cg2677* | - | ABC-type dipeptide/oligopeptide/nickel transport system | EP | 1.38 | 11.57 | $2.48 \times 10^{-06}$ |
| *cg0407* | - | secreted protein | X | 1.37 | 8.82 | $3.86 \times 10^{-03}$ |
| *cg2095* | - | putative membrane protein | S | 1.37 | 10.76 | $2.90 \times 10^{-10}$ |
| *cg1408* | - | putative membrane protein | X | 1.36 | 11.11 | $2.77 \times 10^{-07}$ |
| *cg2096* | - | putative membrane protein | X | 1.36 | 10.24 | $2.31 \times 10^{-08}$ |
| *cg3105* | - | hypothetical protein | X | 1.36 | 9.69 | $1.83 \times 10^{-06}$ |
| *cg1628* | - | hydrolase of the alpha/beta superfamily | R | 1.35 | 11.53 | $4.77 \times 10^{-10}$ |
| *cg1318* | - | DNA repair exonuclease | L | 1.35 | 10.42 | $9.99 \times 10^{-07}$ |
| *cg0841* | - | conserved hypothetical protein | S | 1.34 | 11.54 | $1.96 \times 10^{-06}$ |
| *cg2676* | - | ABC-type dipeptide/oligopeptide/nickel transport system | EP | 1.34 | 9.83 | $6.37 \times 10^{-03}$ |
| *cg2443* | - | permease of the major facilitator superfamily | X | 1.33 | 9.96 | $8.36 \times 10^{-06}$ |
| *cg0465** | - | conserved hypothetical membrane protein | X | 1.30 | 9.08 | $5.33 \times 10^{-03}$ |
| *cg0735* | - | ABC-type transporter | P | 1.28 | 11.35 | $1.82 \times 10^{-07}$ |
| *cg3138* | - | membrane protease subunit | O | 1.27 | 14.29 | $1.03 \times 10^{-04}$ |
| *cg1338* | *thrB* | homoserine kinase | E | 1.25 | 12.98 | $1.03 \times 10^{-04}$ |
| *cg0954* | - | putative secreted protein | X | 1.24 | 8.41 | $1.17 \times 10^{-04}$ |
| *cg3104* | - | ATPase involved in DNA repair | S | 1.22 | 10.18 | $3.23 \times 10^{-07}$ |
| *cg3116** | *cysH* | adenosine-phosphosulfate reductase | EH | 1.20 | 13.90 | $2.70 \times 10^{-05}$ |
| *cg1386* | *fixA* | putative electron transfer flavoprotein | C | 1.18 | 12.19 | $2.02 \times 10^{-10}$ |
| *cg1048* | - | haloacid dehalogenase/epoxide hydrolase family | R | 1.17 | 11.20 | $9.00 \times 10^{-11}$ |
| *cg0742* | - | putative integral membrane protein | X | 1.16 | 9.26 | $9.27 \times 10^{-06}$ |
| *cg1997** | *cglIR* | type II restriction endonuclease | X | 1.16 | 11.29 | $1.09 \times 10^{-06}$ |
| *cg0012* | - | putative transcription regulator protein | KG | 1.14 | 10.93 | $3.96 \times 10^{-06}$ |
| *cg2498* | - | conserved hypothetical protein | X | 1.13 | 10.41 | $5.80 \times 10^{-08}$ |
| *cg1622* | - | ABC-type multidrug/protein/lipid transport system | V | 1.13 | 10.86 | $5.01 \times 10^{-09}$ |
| *cg3141* | *hmp* | flavohemoprotein | C | 1.13 | 10.10 | $2.68 \times 10^{-07}$ |
| *cg1013* | - | hypothetical protein | X | 1.11 | 10.88 | $4.07 \times 10^{-09}$ |
| *cg3082** | - | ArsR-family transcriptional regulator | K | 1.11 | 9.25 | $8.57 \times 10^{-06}$ |
| *cg2391* | *aroG* | phospho-2-dehydro-3-deoxyheptonate aldolase | E | 1.10 | 12.35 | $1.38 \times 10^{-07}$ |
| *cg3140* | *tagA1* | putative DNA-3-methyladenine glycosylase I protein | L | 1.08 | 13.02 | $1.18 \times 10^{-05}$ |
| *cg1018* | - | putative ATP-dependent DNA helicase protein | L | 1.08 | 10.71 | $7.38 \times 10^{-05}$ |
| *cg2960* | - | hypothetical protein | X | 1.08 | 8.64 | $7.25 \times 10^{-04}$ |
| *cg2014* | - | hypothetical protein | D | 1.07 | 10.07 | $1.28 \times 10^{-04}$ |
| *cg3115** | *cysD* | sulfate adenylyltransferase subunit 2 | EH | 1.06 | 13.52 | $8.37 \times 10^{-07}$ |
| *cg0958* | - | secreted protein | X | 1.05 | 9.61 | $1.02 \times 10^{-10}$ |
| *cg1399* | - | permease protein | X | 1.03 | 9.75 | $2.13 \times 10^{-04}$ |
| *cg2232* | *lepB* | putative signal peptidase I transmembrane protein | U | 1.03 | 10.89 | $6.62 \times 10^{-05}$ |
| *cg1376* | *ssuD1* | FMNH2-dependent aliphatic sulfonate monooxygenase | C | 1.03 | 9.46 | $8.54 \times 10^{-07}$ |
| *cg2925* | *ptsS* | enzyme II sucrose protein | G | 1.02 | 10.80 | $2.34 \times 10^{-09}$ |
| *cg0838* | - | helicase family protein | R | 1.01 | 9.78 | $3.05 \times 10^{-04}$ |

| | | | | | | |
|---|---|---|---|---|---|---|
| Genes with decreased expression IB2103 | | | | | | |
| *cg1435* | *ilvB* | acetolactate synthase | EH | -1.00 | 12.18 | $3.49 \times 10^{-05}$ |
| *cg2708* | *msiK1* | ABC-type sugar transport system. ATPase component | G | -1.01 | 13.58 | $3.66 \times 10^{-05}$ |
| *cg1043* | - | thiol-disulfide isomerase and thioredoxin family protein | OC | -1.02 | 9.24 | $5.15 \times 10^{-07}$ |
| *cg2236* | *thiE* | thiamin-phosphate pyrophosphorylase | H | -1.02 | 12.24 | $3.12 \times 10^{-05}$ |
| *cg1546* | *rbsK1* | putative ribokinase protein | G | -1.02 | 9.86 | $1.76 \times 10^{-04}$ |
| *cg2572* | - | conserved hypothetical protein | X | -1.03 | 11.08 | $2.85 \times 10^{-05}$ |
| *cg1300* | *cydB* | cytochrome d terminal oxidase polypeptide | C | -1.03 | 10.18 | $4.13 \times 10^{-05}$ |
| *cg2705* | *amyE* | maltose-binding protein precursor | G | -1.04 | 15.13 | $1.18 \times 10^{-10}$ |
| *cg1832* | - | ABC-type cobalamin/Fe3+-siderophores transport system | P | -1.04 | 8.76 | $4.48 \times 10^{-04}$ |
| *cg1214* | - | cysteine desulfurase or related enzyme | E | -1.05 | 13.09 | $8.46 \times 10^{-07}$ |
| *cg0444* | *ramB* | HTH_3-family transcriptional regulator. | R | -1.05 | 12.26 | $2.05 \times 10^{-07}$ |
| *cg1738* | *acnR* | TetR-family transcriptional regulator | K | -1.05 | 10.16 | $6.58 \times 10^{-06}$ |
| *cg0811* | *dtsR2* | acetyl/propionyl CoA carboxylase | I | -1.06 | 13.31 | $8.09 \times 10^{-10}$ |
| *cg0607* | - | hypothetical secreted protein | X | -1.06 | 10.25 | $5.58 \times 10^{-10}$ |
| *cg0796* | *prpD1* | propionate catabolic protein PrpD | R | -1.08 | 9.76 | $6.77 \times 10^{-05}$ |
| *cg0798* | *prpC1* | (methyl)citrate synthase | C | -1.09 | 11.23 | $1.36 \times 10^{-05}$ |
| *cg2999* | - | putative ferredoxin reductase | R | -1.10 | 11.88 | $2.56 \times 10^{-06}$ |
| *cg1475* | - | conserved hypothetical protein | X | -1.10 | 11.91 | $1.19 \times 10^{-06}$ |
| *cg0118* | *ureG* | urease accessory protein | OK | -1.10 | 10.05 | $4.34 \times 10^{-06}$ |
| *cg0536* | - | putative 5-dehydro-4-deoxyglucarate dehydratase | EM | -1.11 | 10.36 | $8.35 \times 10^{-05}$ |
| *cg1697* | *aspA* | aspartate ammonia-lyase (aspartase) | E | -1.11 | 10.73 | $3.75 \times 10^{-05}$ |
| *cg2840* | *actA* | butyryl-CoA:acetate coenzyme a transferase | C | -1.11 | 15.07 | $8.44 \times 10^{-08}$ |
| *cg2704* | - | ABC-type sugar transport system. permease component | G | -1.12 | 14.25 | $6.36 \times 10^{-09}$ |
| *cg0048* | *ppiA* | peptidyl-prolyl cis-trans isomerase | O | -1.13 | 11.91 | $7.10 \times 10^{-09}$ |
| *cg2477* | - | conserved hypothetical protein | X | -1.14 | 12.47 | $3.05 \times 10^{-06}$ |
| *cg3216* | *gntP* | gluconate permease | GE | -1.14 | 9.72 | $3.10 \times 10^{-05}$ |
| *cg0116* | *ureE* | urease accessory protein | O | -1.15 | 10.20 | $9.30 \times 10^{-07}$ |
| *cg1216* | *nadA* | quinolinate synthetase | H | -1.15 | 14.42 | $1.19 \times 10^{-08}$ |
| *cg1543* | *iunH3* | inosine-uridine preferring nucleoside hydrolase | F | -1.17 | 10.97 | $4.83 \times 10^{-08}$ |
| *cg2157* | *terC* | tellurium resistance membrane protein | P | -1.18 | 11.53 | $6.43 \times 10^{-08}$ |
| *cg2182* | - | ABC-type peptide transport system | EP | -1.23 | 10.45 | $3.28 \times 10^{-06}$ |
| *cg1814* | *carA* | carbamoyl phosphate synthase small subunit | EF | -1.23 | 12.46 | $2.74 \times 10^{-09}$ |
| *cg1224* | *phnB2* | similar to alkylphosphonate uptake operon protein PhnB | S | -1.23 | 9.04 | $2.87 \times 10^{-07}$ |
| *cg1246* | - | conserved hypothetical protein | S | -1.25 | 11.79 | $1.82 \times 10^{-06}$ |
| *cg1087* | - | putative membrane protein | X | -1.25 | 10.03 | $1.74 \times 10^{-05}$ |
| *cg0372* | - | hypothetical protein | X | -1.25 | 10.58 | $1.91 \times 10^{-05}$ |
| *cg1301* | *cydA* | cytochrome d ubiquinol oxidase subunit | C | -1.26 | 9.82 | $2.68 \times 10^{-08}$ |
| *cg2658* | *rpi* | possible phosphopentose isomerase | G | -1.26 | 10.71 | $1.53 \times 10^{-05}$ |
| *cg1817* | *pyrR* | pyrimidine operon attenuation protein | F | -1.27 | 12.47 | $1.27 \times 10^{-07}$ |
| *cg0618* | *fdhF* | putative formate dehydrogenase oxidoreductase | C | -1.27 | 11.41 | $1.89 \times 10^{-08}$ |
| *cg4001** | - | hypothetical protein | X | -1.28 | 12.68 | $3.31 \times 10^{-06}$ |
| *cg0129* | *putA* | proline dehydrogenase | C | -1.28 | 9.47 | $1.47 \times 10^{-06}$ |
| *cg2630* | *pcaG* | protocatechuate dioxygenase alpha subunit | Q | -1.28 | 10.40 | $3.61 \times 10^{-04}$ |
| *cg2836* | *sucD* | succinyl-CoA synthetase alpha subunit | C | -1.30 | 8.37 | $1.96 \times 10^{-10}$ |
| *cg3096* | - | aldehyde dehydrogenase | C | -1.31 | 11.33 | $6.74 \times 10^{-06}$ |
| *cg3282* | - | cation transport ATPase | P | -1.31 | 12.07 | $1.05 \times 10^{-09}$ |
| *cg0216* | - | hypothetical memebrane protein | X | -1.32 | 12.74 | $7.19 \times 10^{-12}$ |
| *cg0386* | *bglS* | beta glucosidase | G | -1.32 | 11.55 | $2.97 \times 10^{-06}$ |
| *cg2875* | - | hypothetical protein | X | -1.34 | 15.09 | $5.04 \times 10^{-06}$ |
| *cg0104* | *codA* | creatinine deaminase | FR | -1.34 | 9.94 | $1.41 \times 10^{-06}$ |
| *cg2183* | - | ABC-type peptide transport system. permease component | EP | -1.34 | 10.81 | $1.61 \times 10^{-05}$ |
| *cg2261* | *amtB* | low-affinity ammonium uptake protein | P | -1.35 | 8.51 | $3.82 \times 10^{-03}$ |
| *cg1247* | - | putative secreted protein | X | -1.36 | 13.02 | $5.07 \times 10^{-08}$ |
| *cg3195* | - | flavin-containing monooxygenase | P | -1.36 | 9.30 | $3.87 \times 10^{-07}$ |
| *cg2429* | *glnA* | glutamine synthetase i | E | -1.38 | 14.01 | $1.21 \times 10^{-07}$ |
| *cg0221* | - | LacI-family transcriptional regulator | K | -1.41 | 13.51 | $1.44 \times 10^{-09}$ |
| *cg2837* | *sucC* | succinyl-CoA synthetase beta subunit | C | -1.42 | 8.81 | $3.95 \times 10^{-06}$ |
| *cg3047* | *ackA* | acetate kinase | C | -1.42 | 10.56 | $4.83 \times 10^{-05}$ |
| *cg1476* | *thiC* | thiamine biosynthesis protein | H | -1.43 | 14.99 | $5.79 \times 10^{-10}$ |
| *cg1813* | *carB* | putative carbamoyl-phosphate synthase subunit | EF | -1.43 | 13.00 | $2.47 \times 10^{-09}$ |
| *cg2260* | *glnK* | nitrogen regulatory protein | E | -1.46 | 10.17 | $4.89 \times 10^{-08}$ |
| *cg0448** | - | conserved hypothetical membrane protein | X | -1.48 | 13.48 | $2.88 \times 10^{-07}$ |
| *cg1109* | *porB* | anion-specific porin precursor | X | -1.48 | 12.98 | $8.10 \times 10^{-07}$ |
| *cg2136* | *gluA* | glutamate uptake system ATP-binding protein | E | -1.49 | 12.83 | $4.74 \times 10^{-09}$ |
| *cg0957* | *fas-IB* | fatty acid synthase | I | -1.52 | 12.32 | $5.64 \times 10^{-08}$ |
| *cg1588* | *argH* | argininosuccinate lyase | E | -1.53 | 9.92 | $3.34 \times 10^{-08}$ |
| *cg0812* | *dtsR1* | acetyl/propionyl-CoA carboxylase beta chain | I | -1.53 | 14.50 | $1.04 \times 10^{-05}$ |

| | | | | | | |
|---|---|---|---|---|---|---|
| *cg2320* | - | putative transcriptional regulator | K | -1.56 | 11.73 | $9.37 \times 10^{-07}$ |
| *cg0049* | - | putative transmembrane protein | R | -1.57 | 12.81 | $5.30 \times 10^{-10}$ |
| *cg3303* | - | putative transcriptional regulator | K | -1.57 | 14.67 | $3.86 \times 10^{-05}$ |
| *cg1582* | *argB* | acetylglutamate kinase | E | -1.58 | 11.34 | $2.26 \times 10^{-02}$ |
| *cg1765* | - | putative transcriptional regulator | K | -1.62 | 13.78 | $1.33 \times 10^{-07}$ |
| *cg1695* | - | putative plasmid maintenance system antidote protein | R | -1.63 | 10.54 | $9.54 \times 10^{-03}$ |
| *cg1586* | *argG* | argininosuccinate synthase | E | -1.64 | 12.94 | $2.71 \times 10^{-08}$ |
| *cg1090* | *ggtB* | putative gamma-glutamyltranspeptidase precursor protein | E | -1.64 | 11.23 | $5.31 \times 10^{-07}$ |
| *cg1833* | - | ABC-type cobalamin/Fe3+-siderophores transport system | P | -1.67 | 8.87 | $1.15 \times 10^{-05}$ |
| *cg3107* | *adhA* | Zn-dependent alcohol dehydrogenase | R | -1.67 | 10.16 | $4.07 \times 10^{-06}$ |
| *cg1718* | - | phospholipid-binding protein | R | -1.68 | 11.60 | $1.30 \times 10^{-02}$ |
| *cg1312* | - | putative membrane protein | X | -1.69 | 11.11 | $4.95 \times 10^{-08}$ |
| *cg1764* | *sufB* | component of an iron-regulated ABC-type transporter | O | -1.75 | 14.04 | $1.84 \times 10^{-07}$ |
| *cg0113* | *ureA* | putative urease (gamma subunit) protein | E | -1.76 | 10.29 | $6.50 \times 10^{-09}$ |
| *cg3048* | *pta* | phosphate acetyltransferase | C | -1.76 | 10.57 | $1.30 \times 10^{-06}$ |
| *cg1785* | *amt* | high-affinity ammonia permease | P | -1.78 | 9.45 | $3.44 \times 10^{-02}$ |
| *cg2181* | - | ABC-type peptide transport system | E | -1.80 | 13.16 | $1.62 \times 10^{-06}$ |
| *cg2138* | *gluC* | glutamate permease | E | -1.81 | 11.72 | $3.20 \times 10^{-09}$ |
| *cg3272* | - | putative membrane protein | S | -1.82 | 11.43 | $2.22 \times 10^{-07}$ |
| *cg2104* | *galE* | UDP-glucose 4-epimerase | M | -1.85 | 10.86 | $2.43 \times 10^{-10}$ |
| *cg1555* | - | superfamily I DNA or RNA helicase | R | -1.86 | 11.30 | $6.59 \times 10^{-03}$ |
| *cg1784* | *ocd* | putative ornithine cyclodeaminase protein | E | -1.87 | 10.13 | $4.36 \times 10^{-06}$ |
| *cg0310* | *katA* | catalase | P | -1.87 | 14.59 | $1.40 \times 10^{-11}$ |
| *cg0253* | - | flavodoxin reductases | C | -1.88 | 10.47 | $3.44 \times 10^{-02}$ |
| *cg1091* | - | hypothetical protein | X | -1.90 | 13.30 | $9.21 \times 10^{-07}$ |
| *cg1760* | - | nifU homolog involved in Fe-S cluster formation | C | -1.92 | 12.64 | $1.18 \times 10^{-06}$ |
| *cg2137* | *gluB* | glutamate secreted binding protein | ET | -1.94 | 12.99 | $6.42 \times 10^{-05}$ |
| *cg1366* | *atpA* | putative ATP synthase alpha chain protein | C | -1.94 | 14.37 | $1.38 \times 10^{-02}$ |
| *cg1737* | *acn* | aconitase | C | -1.99 | 14.53 | $2.94 \times 10^{-10}$ |
| *cg1344* | *narG* | nitrate reductase 2. alpha subunit | C | -2.01 | 11.02 | $3.19 \times 10^{-02}$ |
| *cg2439* | - | hypothetical protein | X | -2.05 | 8.68 | $1.82 \times 10^{-07}$ |
| *cg1783* | *soxA* | sarcosine oxidase - N-terminal fragment | E | -2.11 | 9.73 | $3.90 \times 10^{-06}$ |
| *cg1759* | - | putative metal-sulfur cluster biosynthetic enzyme | R | -2.15 | 12.86 | $2.17 \times 10^{-07}$ |
| *cg0230* | *gltD* | glutamine 2-oxoglutarate aminotransferase | ER | -2.17 | 9.14 | $3.00 \times 10^{-02}$ |
| *cg2560* | *aceA* | isocitrate lyase | C | -2.18 | 10.80 | $1.09 \times 10^{-05}$ |
| *cg1762* | *sufC* | iron-regulated ABC transporter ATPase subunit | O | -2.19 | 13.26 | $6.12 \times 10^{-07}$ |
| *cg1761* | *nifS2* | cysteine desulfhydrase / selenocysteine lyase | E | -2.22 | 12.99 | $7.08 \times 10^{-10}$ |
| *cg1314* | *putP* | proline transport system | ER | -2.25 | 12.60 | $5.57 \times 10^{-12}$ |
| *cg1584* | *argF* | ornithine carbamoyltransferase | E | -2.25 | 11.80 | $1.64 \times 10^{-04}$ |
| *cg1341* | *narI* | respiratory nitrate reductase 2 gamma chain | C | -2.25 | 12.68 | $1.41 \times 10^{-05}$ |
| *cg1585* | *argR* | arginine repressor | E | -2.32 | 12.24 | $1.77 \times 10^{-10}$ |
| *cg2103* | *dtxR* | iron dependent regulatory protein | K | -2.38 | 10.94 | $3.20 \times 10^{-07}$ |
| *cg1580* | *argC* | N-acetyl-gamma-glutamyl-phosphate reductase | E | -2.48 | 13.31 | $4.41 \times 10^{-06}$ |
| *cg0446** | *sdhA* | succinate dehydrogenase a | C | -2.53 | 11.25 | $5.73 \times 10^{-10}$ |
| *cg2636* | *catA1* | catechol 1.2-dioxygenase | Q | -2.56 | 10.19 | $2.12 \times 10^{-05}$ |
| *cg0447** | *sdhB* | succinate dehydrogenase b | C | -2.57 | 13.71 | $6.35 \times 10^{-07}$ |
| *cg0445** | *sdhCD* | succinate dehydrogenase cd | X | -2.57 | 14.01 | $4.06 \times 10^{-09}$ |
| *cg1342* | *narJ* | nitrate reductase delta chain | C | -2.65 | 12.26 | $4.14 \times 10^{-10}$ |
| *cg0952* | - | putative integral membrane protein | S | -2.70 | 12.29 | $5.44 \times 10^{-13}$ |
| *cg0303* | *leuA* | 2-isopropylmalate synthase | E | -2.76 | 13.26 | $2.02 \times 10^{-05}$ |
| *cg0953* | - | Na+/proline. Na+/panthothenate symporters | R | -2.79 | 12.98 | $4.26 \times 10^{-09}$ |
| *cg1583* | *argD* | acetylornithine aminotransferase | E | -2.99 | 12.91 | $9.26 \times 10^{-08}$ |
| *cg1581* | *argJ* | glutamate N-acetyltransferase | E | -3.02 | 13.28 | $5.93 \times 10^{-10}$ |
| *cg2782** | *ftn* | ferritin-like protein | P | -3.36 | 9.47 | $1.01 \times 10^{-07}$ |
| *cg1343* | *narH* | putative respiratory nitrate reductase | C | -3.43 | 12.62 | $1.26 \times 10^{-09}$ |
| *cg1345* | *narK* | putative nitrate/nitrite transporter | P | -3.45 | 12.11 | $4.94 \times 10^{-08}$ |
| *cg3327** | *dps* | starvation-induced DNA protecting protein | P | -3.49 | 13.93 | $2.25 \times 10^{-09}$ |
| *cg0229* | *gltB* | glutamine 2-oxoglutarate aminotransferase l | E | -3.80 | 10.30 | $5.62 \times 10^{-08}$ |
| *cg0569* | - | cation-transporting ATPase | P | -4.89 | 9.41 | $1.07 \times 10^{-07}$ |

[1] Asterisks denote genes that are par tof the DtxR regulon of *C. glutamicum*.
